# Cost-Effective Simulations of Vibrationally-Resolved Absorption Spectra of Fluorophores with Machine-Learning-Based Inhomogeneous Broadening

**DOI:** 10.1021/acs.jctc.2c01285

**Published:** 2023-04-05

**Authors:** Elizaveta F. Petrusevich, Manon H. E. Bousquet, Borys Ośmiałowski, Denis Jacquemin, Josep M. Luis, Robert Zaleśny

**Affiliations:** † Faculty of Chemistry, Wrocław University of Science and Technology, Wyb. Wyspiańskiego 27, PL-50370 Wrocław, Poland; ‡ Institute of Computational Chemistry and Catalysis and Department of Chemistry, University of Girona, Campus de Montilivi, 17003 Girona, Catalonia, Spain; ¶ Nantes Université, CNRS, CEISAM UMR 6230, F-44000 Nantes, France; § Faculty of Chemistry, Nicolaus Copernicus University, Gagarina Street 7, PL-87-100 Toruń, Poland; ∥ Institut Universitaire de France (IUF), F-75005 Paris, France; ⊥ Institute of Computational Chemistry and Catalysis and Department of Chemistry, University of Girona, Campus de Montilivi, 17003 Girona, Catalonia, Spain

## Abstract

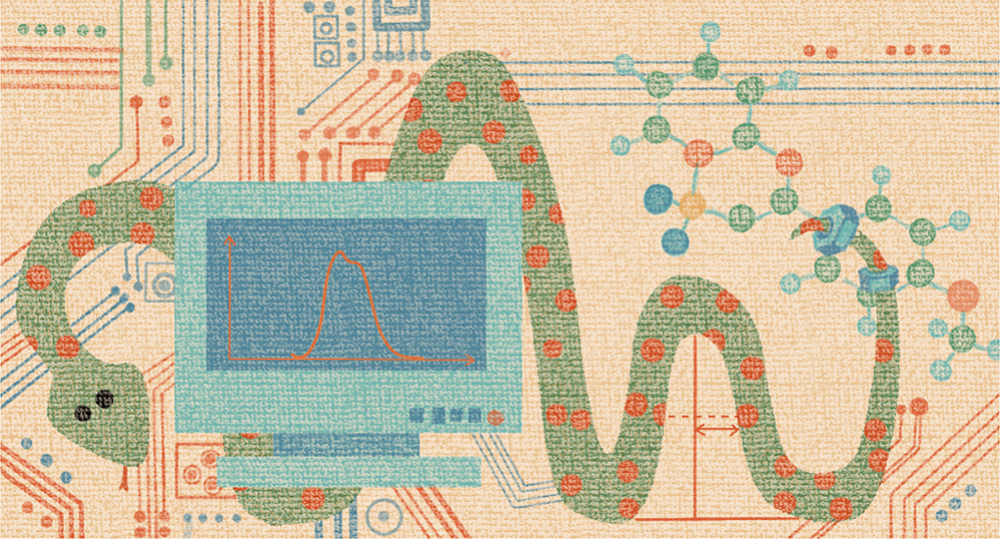

The results of electronic and vibrational structure simulations are an invaluable support for interpreting experimental absorption/emission spectra, which stimulates the development of reliable and cost-effective computational protocols. In this work, we contribute to these efforts and propose an efficient first-principle protocol for simulating vibrationally-resolved absorption spectra, including nonempirical estimations of the inhomogeneous broadening. To this end, we analyze three key aspects: *(i)* a metric-based selection of density functional approximation (DFA) so to benefit from the computational efficiency of time-dependent density function theory (TD-DFT) while safeguarding the accuracy of the vibrationally-resolved spectra, *(ii)* an assessment of two vibrational structure schemes (vertical gradient and adiabatic Hessian) to compute the Franck–Condon factors, and *(iii)* the use of machine learning to speed up nonempirical estimations of the inhomogeneous broadening. In more detail, we predict the absorption band shapes for a set of 20 medium-sized fluorescent dyes, focusing on the bright *ππ*^★^ S_0_ → S_1_ transition and using experimental results as references. We demonstrate that, for the studied 20-dye set which includes structures with large structural variability, the preselection of DFAs based on an easily accessible metric ensures accurate band shapes with respect to the reference approach and that range-separated functionals show the best performance when combined with the vertical gradient model. As far as band widths are concerned, we propose a new machine-learning-based approach for determining the inhomogeneous broadening induced by the solvent microenvironment. This approach is shown to be very robust offering inhomogeneous broadenings with errors as small as 2 cm^–1^ with respect to genuine electronic-structure calculations, with a total CPU time reduced by 98%.

## 1 Introduction

Fluorescent dyes are very popular in many fields, including medicine and biochemistry, owing to their in-demand sensitivity and selectivity. Although there is a large number of fluorophore families, boron-carrying compounds remain in the limelight since they generally offer highly valuable optical properties. The most popular boron-carrying fluorescent core is undoubtedly the boron-dipyrromethene (BODIPY) chromogen. Based on the BODIPY core, an astonishingly diversified panel of dyes was obtained, and several are used as commercial fluorescent probes.^1−5^ However, the BODIPY derivatives are not flawless, e.g., they typically possess relatively small Stokes shift, which impedes applications in several fields. However, there are several other classes of difluoroborate dyes that are as efficient emitters as BODIPYs yet present distinct structural features allowing alleviation of some of the BODIPYs’ limitations (see a review by the Strasbourg group^6^).

**Scheme 1:**
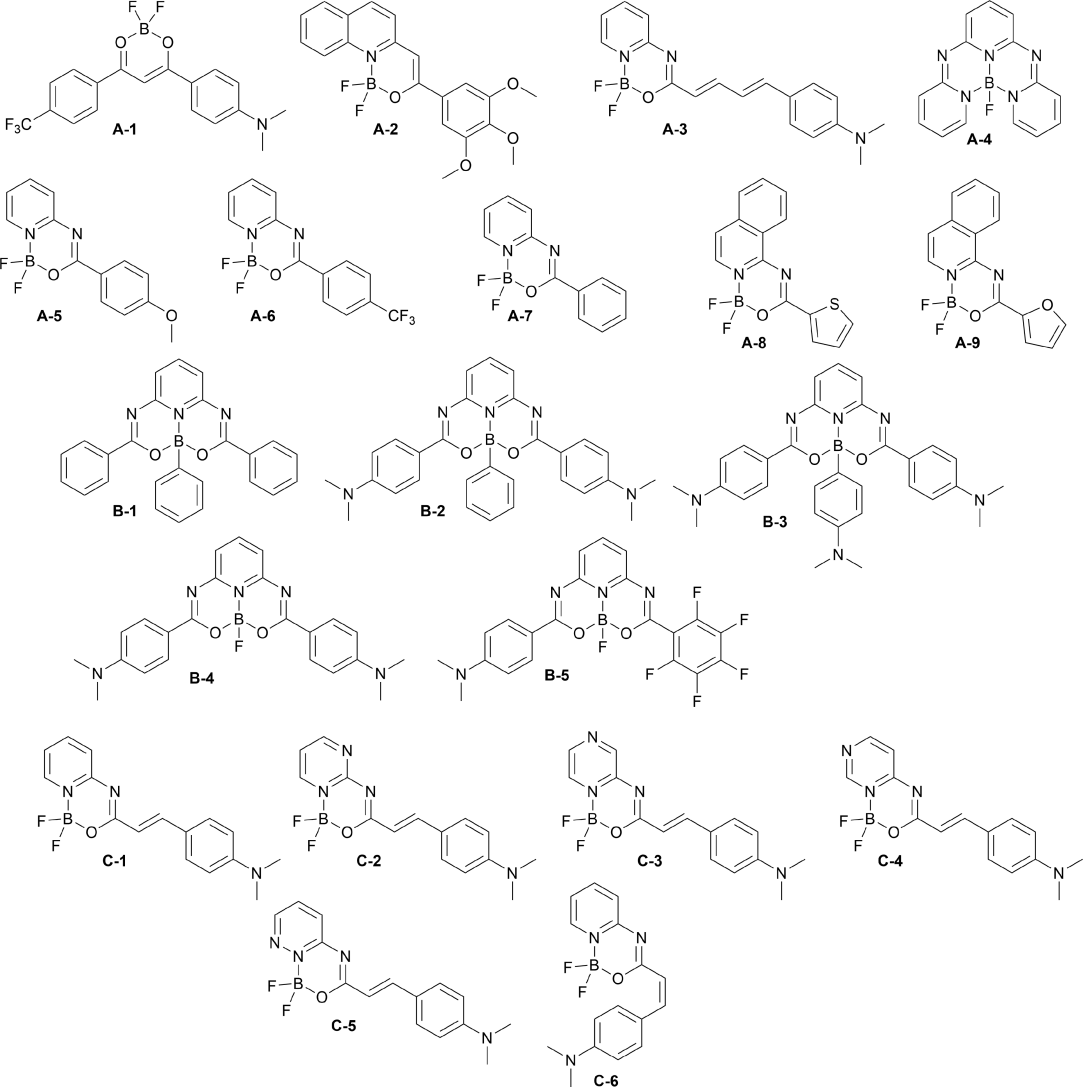
Set of Dyes Studied in This Work

Of course, any efficient application of fluorescent dyes requires that the photophysical properties of the emitter are thoroughly characterized. To this end, electronic absorption and emission spectroscopies are widely used, with such measurements being typically performed in solution. Nonetheless, the interpretation of the experimental data might be far from straightforward since the recorded spectral signatures are often stemming from a complex blend of effects. Challenging cases include *(i)* the presence of significant vibronic couplings resulting in unusual band shapes, *(ii)* a near degeneracy between two or more electronic excited states leading to overlapping absorption bands, and *(iii)* an unexpected magnitude of the inhomogeneous broadening coming from solute–solvent interactions. These effects can be, at least partially, rationalized using electronic and vibrational structure calculations. Obviously, to fully exploit the potential of first-principle simulations, it is mandatory to select a reliable and robust protocol, ideally not requiring extensive computational resources. There is a vast computational spectroscopy literature covering a wide palette of aspects, including *(i)*–*(iii)* and suggesting strategies to select the most appropriate models for treating different types of states and compounds.^7−69^ However, by and large, published works either treat these aspects *separately* or provide a detailed analysis for a selected dye or a small set of closely related compounds. The aim of the present contribution is to propose an efficient protocol to overcome some of these limitations and discuss the accuracy and reliability of electronic and vibrational structure methods for simulating vibrationally-resolved electronic absorption spectra, including nonempirical estimations of the inhomogeneous broadening. In this context, we have chosen a set of 20 boron-containing fluorescent dyes of medium sizes yet presenting significant structural variations (Scheme 1). The specific goals of this work are *(i)* adequately choosing a density functional approximation (DFA) so as to retain a low computational cost while guaranteeing accurate simulations of the vibrationally-resolved electronic absorption spectra, *(ii)* assessing various vibrational structure theories to compute Franck–Condon factors, and *(iii)* applying a machine learning model to speed up the nonempirical estimations of the inhomogeneous broadening.

## 2 Computational Details

### 2.1 DFA Selection

The selection of DFAs for simulating the vibrationally-resolved spectra relies on the vibrational reorganization energy as the metric (see the next Section)^62^ and is achieved in the gas phase using the resolution-of-identity (RI) second-order coupled-cluster CC2 model^65,70^ (RI-CC2) as the benchmark. The considered palette of DFAs encompasses the following: BLYP,^71,72^ B3LYP,^73^ PBE0,^74,75^ MN15,^76^ BH&HLYP,^77^ M06-2X,^78^ CAM-B3LYP,^79^ ωB97X,^80^ ωB97X-D,^81^ and LC-BLYP,^82^ as well as two optimally tuned variants of the latter, namely LC-BLYP-OT(J) and LC-BLYP-OT(α).^83^ The OT(J) variant employs the optimized value of the range-separation parameter which fulfills the DFT version of Koopmans’ theorem (i.e., Janak’s theorem), while the OT(α) variant employs the range-separation parameter reproducing the CCSD(T) second hyperpolarizabilities.^83^ The ground-state geometries are first optimized using each of the above listed DFAs and subsequently used in evaluation of both the ground-state Hessians and the excited-state gradients. All these calculations were performed using the cc-pVDZ atomic basis set.^84^ The so-called *ultrafine* pruned integration grid is selected, and all geometry optimizations use the *tight* convergence as implemented in the Gaussian 16 program.^85^ The corresponding RI-CC2 model calculations are performed with the TURBOMOLE 6.5 program.^86^

### 2.2 Vibrationally-Resolved Electronic Spectra Calculations

The DFAs selected in the first step are subsequently applied for calculating the vibrationally-resolved electronic spectra in solution, the solvent being modeled using the integral-equation-formalism (IEF) version of the polarizable continuum model (PCM), considering chloroform as medium.^87,88^ Both the geometry optimizations and Hessian calculations in the ground and first electronic excited states are performed with the aug-cc-pVDZ atomic basis set.^84^ The pruned *superfine* grid available in Gaussian is applied in these calculations. We computed the vibrationally-resolved spectra applying both the vertical gradient (VG) and adiabatic Hessian (AH) potential energy surface models.^12^ These calculations are performed with the FCclasses 3.1 code adopting the harmonic approximation.^9,12,89−91^ In what follows, we will refer to the vibronic spectra as simulated vibrationally-resolved electronic spectra considering only the Franck–Condon terms (i.e., without taking into account the higher-order terms due to the coupling between electronic and vibrational degrees of freedom). In this work, beyond the well-known VG and AH schemes, we also use another variant termed as the “displaced adiabatic hessian” (dAH) method. In the dAH model, one uses the same excited-state geometry as estimated by the VG model, assuming perfect quadratic PES to describe the excited state. Subsequently, the excited-state Hessian is computed at the VG-predicted geometry, which obviously is different from that used in the AH method, thus justifying the “displaced AH” name. This method is also known as the Adiabatic Hessian After Step^68^ (AHAS), but we use different labels to highlight that we used an in-house code to perform such calculations. If imaginary frequencies appear in the excited-state Hessian, then we follow the standard FCclasses routine: we turn them into real numbers by taking their module. Both the Time-Independent (TI, with number of integrals up to 10^12^) and Time-Dependent (TD) line shape formalisms are employed, and the Franck–Condon approximation is selected since we treat strongly dipole-allowed transitions. For all calculations, the temperature is set to 298 K, and both Cartesian and internal coordinates are employed to get the vibrational normal modes. Gaussian broadening functions are used to simulate the inhomogeneous broadening.

### 2.3 Estimation of the Inhomogeneous Broadening

Inhomogeneous broadenings corresponding to the distribution of electronic excitation energies due to the variable local microenvironment of the dyes are determined based on rigid-body molecular dynamics (RB-MD) simulations for dyes **B-1**–**B-5** (Scheme 1) performed with the NAMD program.^92^ In order to allow for comparisons with experiments we perform the RB-MD simulations for these five compounds in two solvents (chloroform and acetonitrile). The use of frozen coordinates for the dyes’ nuclei is justified since we wish to avoid double-counting of vibrational contributions during convolutions with stick vibronic spectra. In more detail, the geometries are first optimized at the CAM-B3LYP/cc-pVDZ level considering the two solvents as modeled by PCM. Atomistic models using 40 × 40 × 40 Å boxes with the dyes surrounded by chloroform or acetonitrile molecules are next used. The solvent density in these boxes is close to the solvent density at room temperature (1.479 g/mL for chloroform and 0.783 g/mL for acetonitrile). Since we use rigid-body MD, only partial charges are required for the description of the chromophore. For parametrizing the force field, we calculated partial atomic CHELPG charges of both the dye and solvent molecules at the CAM-B3LYP/cc-pVDZ level of theory considering the equilibrium geometry. Leonard-Jones parameters taken from the CHARMM force field^93,94^ are used to describe the dyes. For the description of chloroform (acetonitrile), the force field parameters obtained by Dietz and Heinzinger^95,96^ (implemented into CHARMM)^94^ are selected. The system is minimized during 30000 steps (1 step = 2 fs) followed by constant temperature NVT dynamics for 12 ns at 300 K using a Langevin thermostat with periodic boundary conditions. For each molecule, 10,000 solute–solvent configurations (with a spacing step of 2 ps) are extracted for each solvent from the resulting trajectory for subsequent electronic-structure calculations with Gaussian 16.^85^ For the discrete representation of the solvent, the electrostatic embedding (EE) approach is used. In that case geometries are taken from snapshots of the MD simulations, and partial charges are the same as within MD simulations.

Based on the distribution of the vertical transition energies to the S_1_ state, the standard deviation is estimated using a Gaussian-like distribution. As the S_0_ → S_1_ transition exhibits intramolecular charge transfer character in a few compounds of the **B** series, we selected the CAM-B3LYP/cc-pVDZ level of theory for these calculations. It was demonstrated that this range-separated hybrid functional handles well charge-transfer excitations.^63^ Subsequently, these values of standard deviations are used as parameters of Gaussian line profiles used to broaden the stick vibronic spectra. In this work, we also use the machine learning (ML) model to lower the computational costs corresponding to the determination of the inhomogeneous broadening. We apply the available data for the set of 10,000 solute–solvent configurations per molecule/solvent (vertical excitation energies obtained using TD-DFT, distribution of solvent molecules around a rigid solute with corresponding charges) and employ the Kernel Ridge Regression model as implemented in the QML toolbox.^97^ ML calculations employ a newly proposed one-dimensional fingerprint based on the Coulomb matrix^98^ (see the next Section for details). The training is performed for 200 randomly selected snapshots from the full 10,000 set for each solute/solvent combination. Once the model is trained, the predictions are performed for the remaining 9,800 data points.

## 3 Results and Discussion

As stated above, the set of molecules (Scheme 1) considered encompasses a wide range of structural features, i.e., *(i)* a rigid molecule (**A-4**), *(ii)* molecules with an unsubstituted phenylene moiety having significant rotational freedom (**A-7**, **B-1**, **B-2**), *(iii)* compounds with a donor–acceptor substituted phenylene moiety (**A-1**, **B-2**, etc.), *(iv)* derivatives with a vinylene linker that are prone to photoisomerization, which dramatically tunes the spectral signatures^99^ (**C** series), and *(v)* a molecule with two vinylene linkers^100^ (**A-3**). The structural variability present in the set leads to a wide range of charge-transfer properties for the lowest electronic excited states, as illustrated by the variable experimental absorption band widths (see Figures S1–S3 in the SI). The SI also contains a concise description of other experimental data available, including the synthesis of newly obtained dyes present in the set.

### 3.1 Choosing a DFA for the Simulations of Vibrationally-Resolved Electronic Absorption Spectra

**Figure 1:**
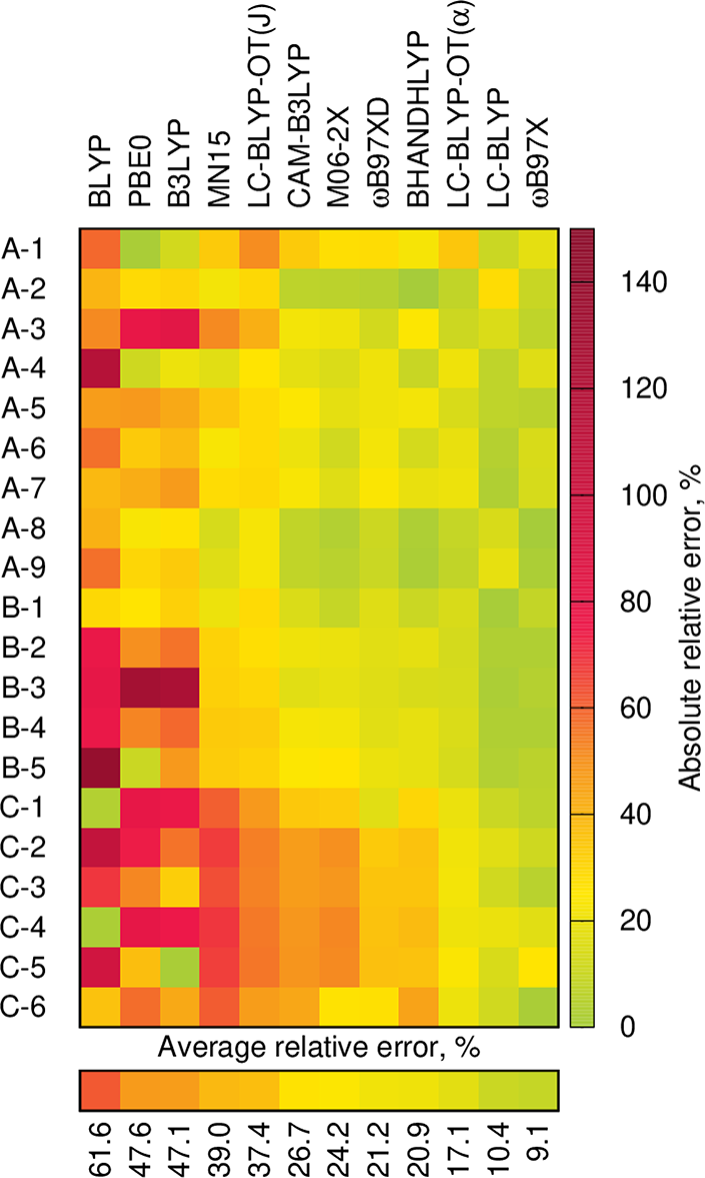
Unsigned relative errors in vibrational reorganization energies obtained with various DFAs using CC2 data as reference. All data are in the gas phase; see the SI for details.

There is a vast literature devoted to the accuracy of QM methods for the calculations of vertical excitation energies (see the Introduction). There are indeed reviews that provide guidelines to select a DFA for transition energies. Given the scope of the present work, it should be mentioned that several DFAs offer satisfactorily accurate electronic absorption spectra, i.e., the average errors can be ca. 0.2 eV or smaller,^101−104^ yet fluoroborates are particularly challenging.^43,105^ In contrast to calculations of excitation energies, the reliability of various DFAs for simulating the vibrationally-resolved spectra using density-functional theory was less explored.^35,47,61,62,106^ This is certainly due to the large computational cost associated with vibronic calculations, especially for post-Hartree–Fock electronic structure theories required to compute the theoretical benchmark values. Two take-home messages, confirmed by several research groups, are that (*i*) DFAs suitable for the accurate predictions of absorption band positions are not necessarily the best for the band shapes and *vice versa*; (*ii*) range-separated (RS) DFAs demonstrate overall good predictive power as far as distributions of Franck–Condon factors are concerned,^62^ albeit there are striking exceptions;^106^ (*iii*) the selected approach used to build the vibrational space (e.g., VG or AH) significantly impacts the computed band shapes;^12,107^ hence, the most accurate DFA(s) cannot *a priori* be selected independently of the vibronic approach. These challenges in quantifying the DFA errors in predicting the vibrational fine structure of absorption bands motivated some of us to propose a simple quantitative metric based on vibrational reorganization energy, λ_vib_, as a tool for selecting an appropriate DFA.^62^ This reorganization energy reads
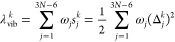
1where *k* labels the excited state, ω_*j*_ is the vibrational frequency, and *s*_*j*_^*k*^ is the Huang–Rhys factor related to the *j*^th^ normal mode dimensionless displacement Δ_*j*_^*k*^ between the electronic state *k* and the ground-state minima. One can use a relatively robust wave function method (e.g., RI-CC2 combined with medium-sized basis sets) to determine reference λ_vib_ values in the VG approach, which can next be used to screen a wide palette of DFAs applied with the same vibronic model. The DFA delivering the smallest error in λ_vib_ can subsequently be selected to perform sophisticated vibronic calculations. The effectiveness of this simple strategy was demonstrated by comparing the simulated vibrationally-resolved electronic absorption spectra of four chromophores with known experimental spectra.^62^ In this work, we rely on the above metric, using RI-CC2 data as reference, to select the most accurate DFAs based on gas-phase VG analyses. The wide palette of DFAs used for this propose contains the following: one semilocal (BLYP), a series of global hybrids (B3LYP, PBE0, MN15, BHandHLYP, M06-2X), and range-separated hybrids (CAM-B3LYP, ωB97X-D, ωB97X, LC-BLYP, and optimally tuned LC-BLYP-OT(J) and LC-BLYP-OT(α)). To our knowledge, the two latter optimally tuned DFAs as well as MN15 (except ref (108)) have not been previously benchmarked for the simulation of vibrationally-resolved spectra, although other types of optimally tuned functionals were used to that end.^109^ For the present benchmark, we determine Δ_*j*_^*k*^ using excited-state gradients and ground-state Hessian, which is an efficient approach.^62^Figure 1 displays the unsigned relative errors in vibrational reorganization energy of the 12 above-mentioned DFAs with respect to CC2 considering all 20 compounds, and λ_vib_ values are given in Table S2 and Figure S4 in the SI. In Figure 1, the DFAs are ranked by their average error. Clearly the three range-separated hybrids, namely, ωB97X (ave. 9.09%, max. 25.91%), LC-BLYP (ave. 10.37%, max. 28.36%), and LC-BLYP-OT(α) (ave. 17.08%, max. 34.85%), provide the most accurate results. One can also notice the much worse performance of MN15 (39.02% error) as compared to one of its predecessors, M06-2X (24.17% error). This is rather disappointing since MN15 was shown to provide very accurate vertical excitation energies and excited-state dipole moments of similar compounds.^110^ More generally, the results shown in Figure 1 are similar to those previously obtained for another set of boron difluoroborates.^62^ A comparison between the vibrationally-resolved spectra computed using the VG vibronic approach on the basis of “the most accurate” (ωB97X) and “the least accurate” (BLYP) DFA is provided in Figure S7 in the SI, while Figure S8 also shows the comparison of MN15 (“mediocral” DFA) with ωB97X. As expected, the vibrational fine structure of the absorption band crucially depends on the magnitude of vibrational reorganization energy (see also Figures S33 and S34).

### 3.2 Validation of VG and AH Models

For assessing the VG and AH methods, both ωB97X and LC-BLYP-OT(α) are selected and compared to experiment for the full series of compounds. The former DFA was considered since it delivers the smallest average error; the latter was considered since it provides highly accurate second hyperpolarizabilities,^83^ two-photon absorption strengths correlating with their CC2 counterparts,^111^ and behaves well for the band topologies, as demonstrated above. This could pave the way to the modeling of the band topologies of two-photon absorption bands, that are related to the imaginary part of the second hyperpolarizability. Figures S9–S11 in the SI provide a comparison between the simulated absorption band corresponding to the S_0_ → S_1_ transition with the two selected DFAs for all three sets of studied dyes (**A**, **B**, and **C**) in chloroform. The spectra simulated using these two functionals are very similar which comes as no surprise given their similar λ_vib_ values.

Let us now turn to a comparison with experiment. We recall that both VG and AH calculations require some preparatory electronic-structure calculations, i.e., the VG model requires the evaluation of the ground-state Hessian and the excited-state gradient at the ground-state geometry, whereas the AH model additionally needs the excited-state Hessian at the corresponding optimal structure. The latter calculations are computationally expensive for molecules composed of more than several dozens of atoms, even at the TD-DFT level. For both the VG and AH calculations, we use the time-independent and time-dependent formalisms; yet for the majority of compounds, the former leads to a low recovery factor of the Franck–Condon spectra if the number of integrals is smaller than 10^12^. Therefore, we include and describe below the results obtained within the time-dependent model only. The full set of vibrationally-resolved spectra obtained using both VG and AH models with the two selected DFAs can be found in Figures S9–S34 in the SI. We underline that Figures S18, S20, and S22 show the results obtained with LC-BLYP-OT(α), assuming a Gaussian profile for the inhomogeneous broadening with a half width at half-maximum (HWHM) arbitrarily set to 100 cm^–1^ to allow consistent comparisons in the series. This comparison allows us to conclude that the AH model, for more than a half of molecules, predicts much longer vibrational progressions than the VG one, which is a known trend.^107^ In fact, in a few cases, one even observes unphysically large band broadenings with the AH model, although this effect can be in part alleviated by using internal rather than Cartesian coordinates (see Figures S12–S17).^112^ We note that the literature points out that in vertical models, the internal coordinates are almost systematically superior to their Cartesian counterparts. On the other hand, in adiabatic models, the optimal coordinate system is less clear-cut and hence depends on the investigated systems, since in practice two sets of internal coordinates are used in the AH model, and the connection between these two is not always straightforwardly established.^112^ For the present set, there are only a few cases where agreement between VG and AH models is satisfactory. These conclusions also hold for the spectra obtained with ωB97X, as can be seen from Figures S24, S25, and S26.

**Figure 2:**
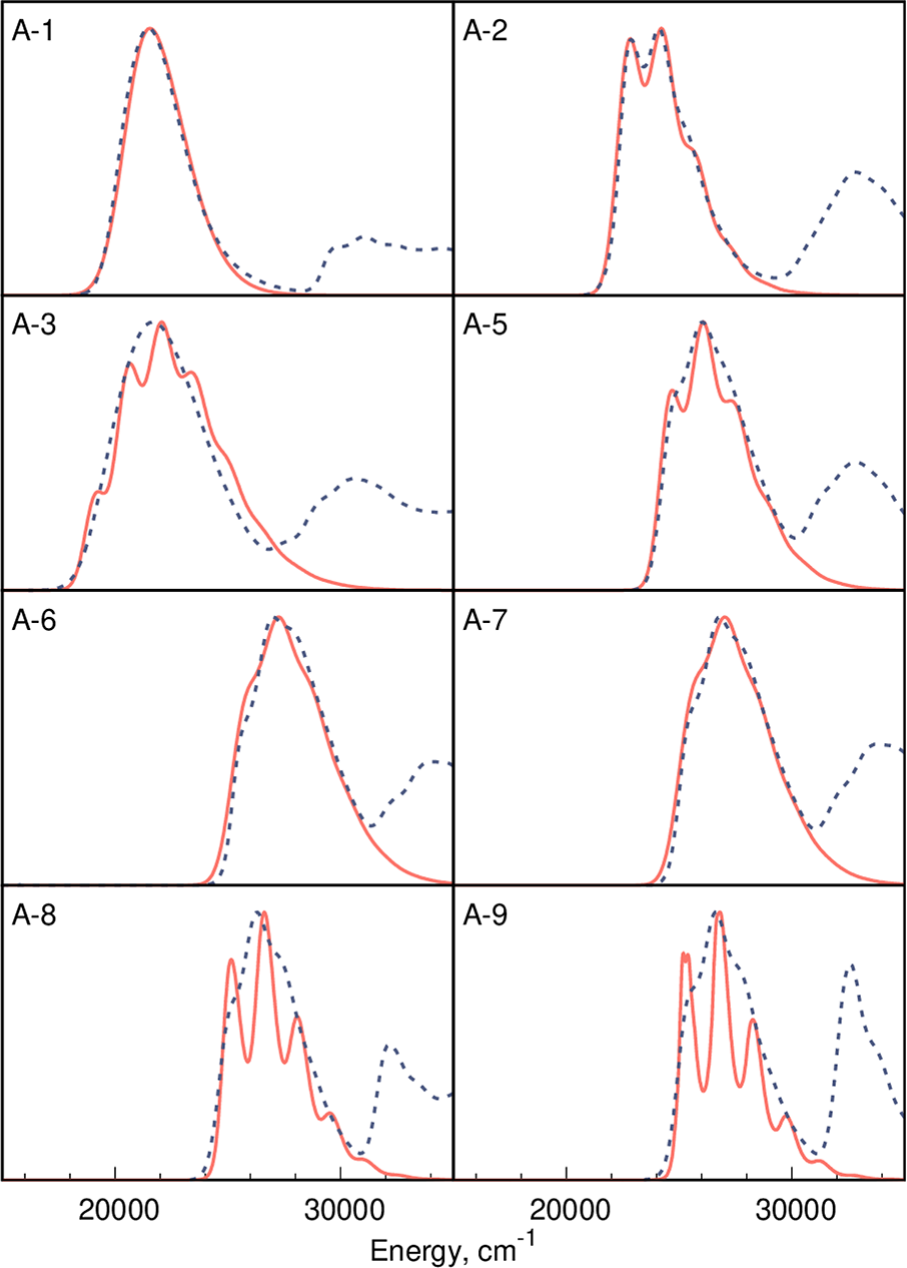
Comparison between the LC-BLYP-OT(α) results relying on the VG vibronic model (red solid line) and the experimental data (blue dotted line) for dyes of series **A**. The simulated spectra use an empirical Gaussian broadening reproducing the measured band width.

**Figure 3:**
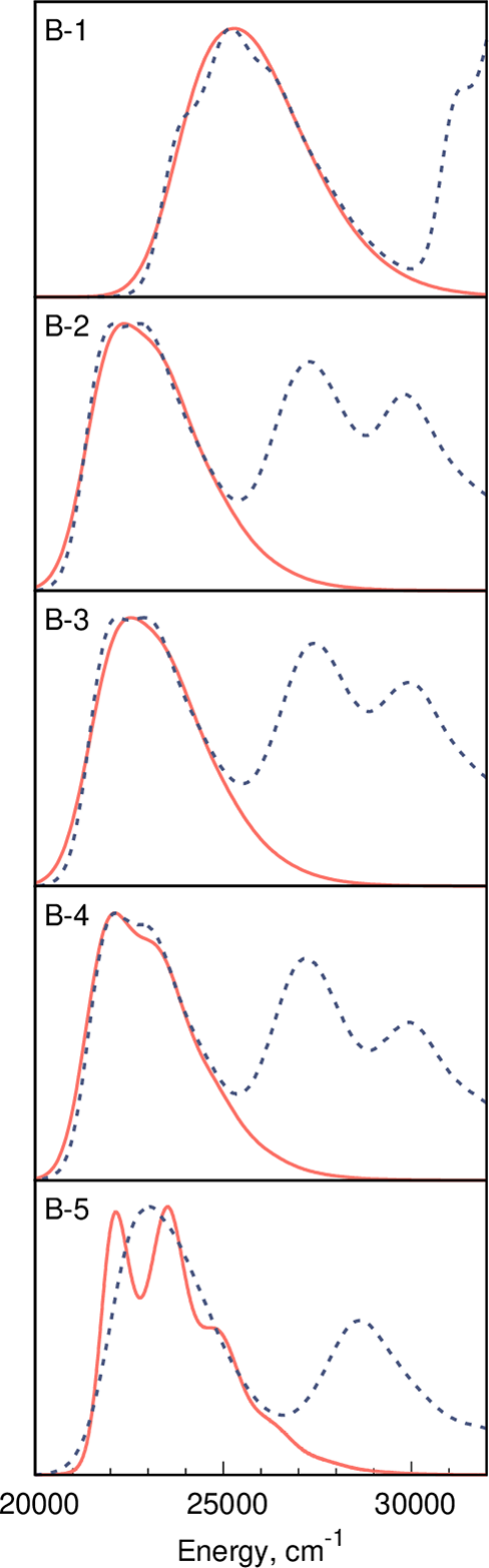
Comparison between the LC-BLYP-OT(α) and the experimental spectra for compounds of series **B**. See the caption of Figure 2 for more details.

**Figure 4:**
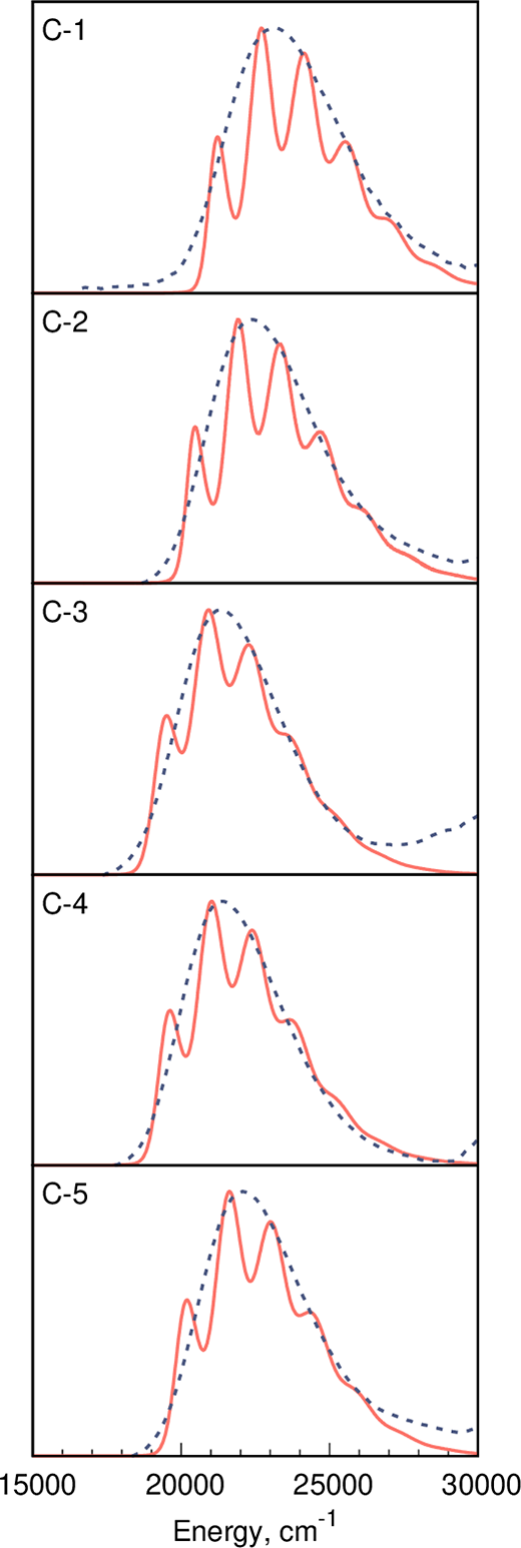
Comparison between the LC-BLYP-OT(α) and the experimental spectra for compounds of series **C**. See the caption of Figure 2 for more details.

Given the observed significant differences between band profiles computed with the VG and AH models, we attempted to assess their accuracy using experimental data as reference (see Figures S1–S3 in the SI). Let us start with a discussion of the VG results. The simulated spectra were obtained by tuning the inhomogeneous broadening so as to fit the experimental band widths. The LC-BLYP-OT(α) simulated spectra obtained in this way are displayed in Figures 2–4. For dyes of the **A** group, the VG model reproduces experimental band shapes with high accuracy, i.e., the relative intensities of the 0–0/0-*n* peaks are nicely recovered. For the fluorophores of both the **B** and **C** series, the experimental spectra are mostly structureless, and obtaining definitive conclusions is therefore harder. Figures S27, S29, and S31 present the comparison of spectra simulated with the AH model with experimental data. Due to long vibrational progressions with AH, no fitting was performed, and very small values of inhomogeneous broadening were used to simulate the spectra. By and large, these comparisons indicate that the AH model is simply poorly predicting the distributions of the FC factors. In order to better understand the underlying reasons for this poor performance, calculations of the absorption spectra using its variant hereafter denoted as the displaced-AH model (dAH) were performed. In dAH, the VG excited-state geometry minimum, that is the minimum obtained using the excited-state gradient and assuming quadratic potential energy surface derived from the ground-state Hessian, is used to compute the excited-state Hessian allowing next to perform AH-like calculations. Figures S19, S21, and S23 demonstrate that this approach, which differs from AH only in the selected excited-state geometry (and corresponding excited-state Hessian), alleviates some of the unphysically long vibrational progressions obtained with AH. This suggests that the differences between ground- and excited-state geometries are too large to fit a harmonic model, thus explaining the poor performance of the AH model in some cases.

### 3.3 Nonempirical Estimations of Inhomogeneous Broadening

**Figure 5:**
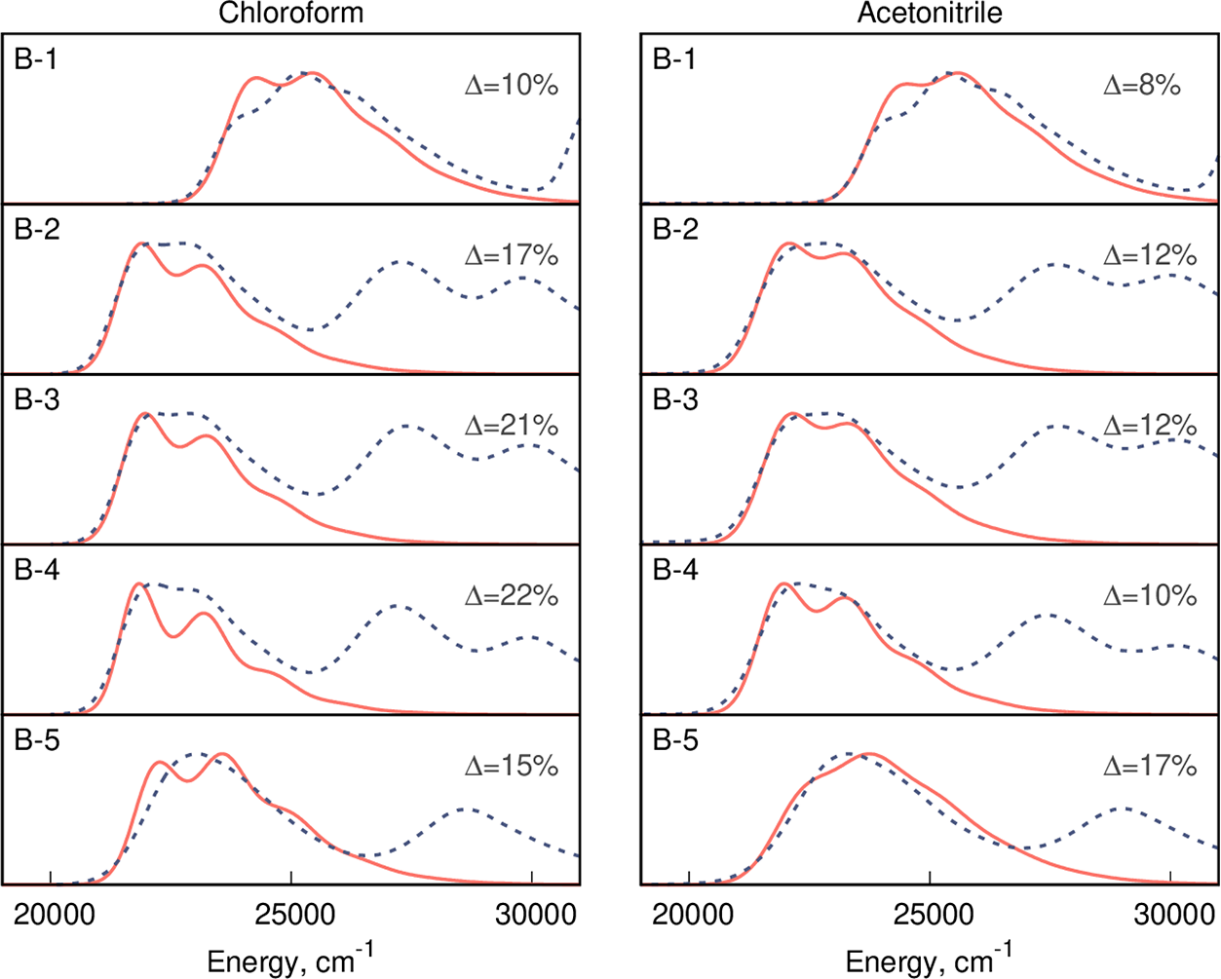
Comparison between the simulated (red solid line) and the experimental (blue dotted line) spectra for five **B** derivatives. The simulations were performed using the VG method and the LC-BLYP-OT(α) DFA with an inhomogeneous broadening estimated using rigid-body MD. See the text for details.

Given the success of the cost-effective VG model in predicting accurate vibrationally-resolved electronic spectra, applying either an arbitrarily chosen inhomogeneous broadening or fitting the broadening to match the measured band widths, we go a step further in this Section. Specifically, as proposed by some authors, the inhomogeneous broadening can be estimated based on rigid-body molecular dynamics simulations followed by electronic-structure calculations. In such MD approaches, the solvent representation can be achieved using various embedding schemes.^51,57,113−117^ Even though we are well aware that EE is a simpler and less accurate approach than more sophisticated embedding schemes relying on higher-order electrical moments, it can be advantageously applied with many electronic structure codes. With efficiency in mind, EE was chosen for our rigid-body MD simulations here. In more detail, we perform MD simulations for five dyes of the **B** series in two solvents with largely different dielectric constants, namely chloroform (ϵ = 4.7) and acetonitrile (ϵ = 35.7). We next select 10,000 snapshots from each rigid-body MD trajectory to carry out TD-DFT calculations; that is we perform a total of 100,000 TD-DFT vertical energy determinations, in order to estimate the inhomogeneous broadening corresponding to S_0_ → S_1_ transition. These broadenings are next used in the VG calculations allowing a comparison of the computed and experimental band shapes. The results are shown in Figure 5 and demonstrate that the cost-effective EE approach leads to errors in the final fwhm pleasingly smaller than 25% (average errors of 17% in chloroform and 12% in acetonitrile). The overall visual agreement between theory and experiment is also satisfactory.

**Figure 6:**
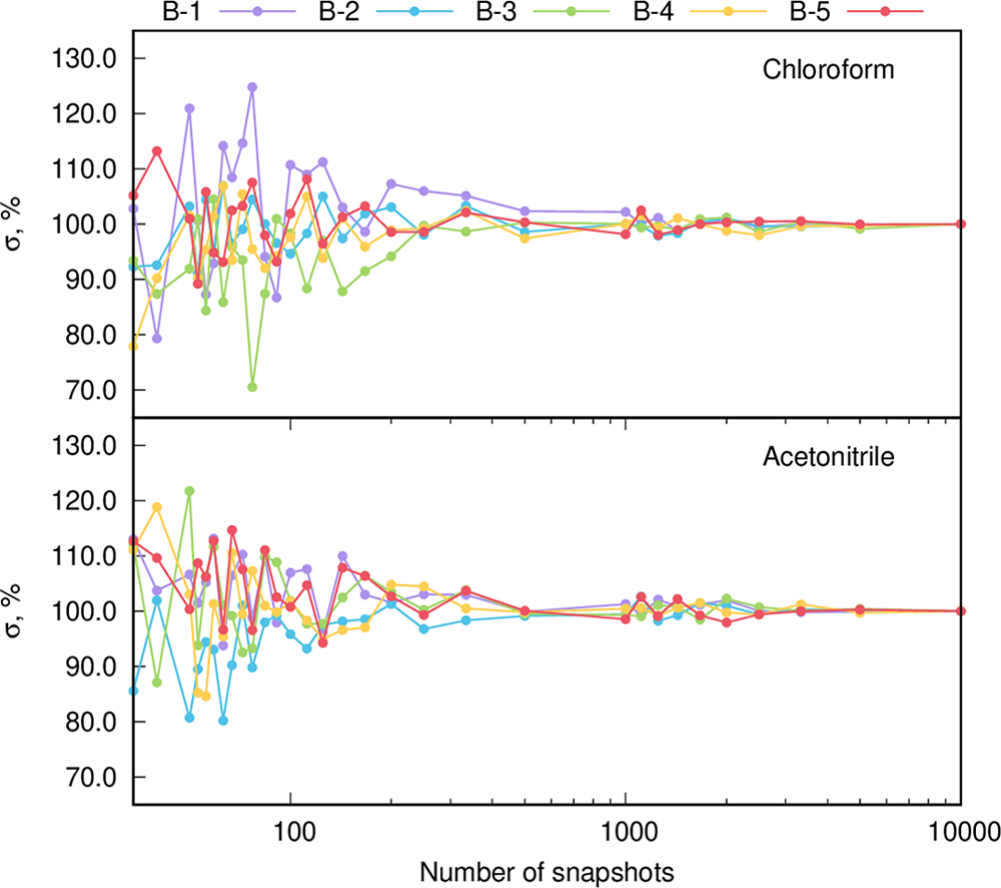
Convergence of the standard deviation corresponding to the distribution of vertical excitation energy with respect to the number of snapshots for the five considered dyes and two solvents.

**Figure 7:**
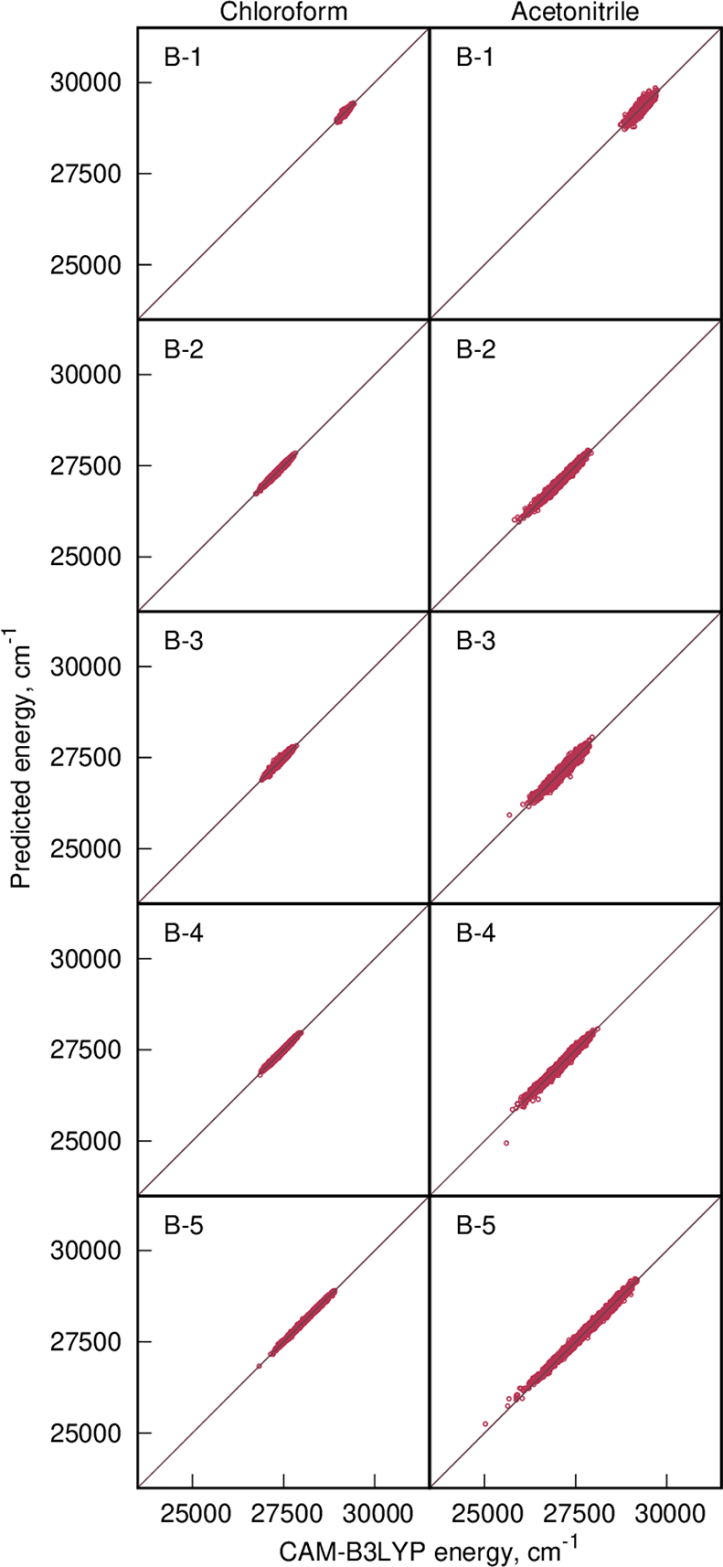
Scatter plot by CAM-B3LYP of the calculated (*X*-axis) and predicted (based on a machine learning model; *Y*-axis) values of vertical excitation energy for five dyes in two solvents.

Figure 6 demonstrates the convergence of the standard deviation corresponding to distribution of vertical excitation energies as a function of the number of (statistically uncorrelated) snapshots from MD simulations. It turns out that at least 2,000 snapshots are required to obtain fully converged values. Even though the EE approach is inexpensive, the computational cost associated with such a large number of TD-DFT calculations remains significant. In an attempt to reduce this cost, we employ a machine learning model relying on Kernel Ridge Regression (KRR, with Gaussian kernel) and propose the following one-dimensional Coulomb-type fingerprint
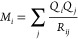
2where *M*_*i*_ represents the interaction between the dye’s atom *i* and all solvent atoms *j*; *Q*_*i*_ and *Q*_*j*_ are respectively the net point charges of the dye atom *i* and solvent atom *j*; and *R*_*ij*_ is the distance between the solvent atom *j* and dye atom *i*. The above fingerprint is derived from the Coulomb matrix (CM) proposed by Rupp et al.;^98^ however, we neglect intradye, intrasolvent as well as intersolvent atom–atom interactions. In doing so, we only probe the fluctuations of the local microenvironment of the atomic sites of the dye. This approach advantageously makes the size of the fingerprint equal to the number of atoms in the dye, whereas applying CM fingerprints in the present case would lead to matrices containing more than six million elements. With the above fingerprint, we used 200 statistically uncorrelated snapshots for each dye to train the ML model (independently for each dye and each solvent). In more detail, for each snapshot, we use the excitation energy, as predicted by the TD-DFT approach, and net charges corresponding to the dye and solvent molecules (see eq 2). Based on such a trained model, the distribution of the vertical excitation energies for the remaining 9,800 snapshots can be predicted. These values obtained thanks to the trained machine-learning model can be compared with excitation energies determined using TD-DFT (available for all 10,000 snapshots) to assess the performance of the former. Based on the results shown in Figure 7 and Table 1, we draw two major conclusions for our set of five molecules and two solvents. First, the average error on the predicted excitation energy is trifling: it amounts to 26.2 cm^–1^ (0.003 eV) only. Second, our ML protocol predicts the standard deviation (σ^inh^) corresponding to the distribution of the excitation energies even more accurately than the average error with respect to the reference results computed using TD-DFT, i.e., the error of the ML predicted σ^inh^ is 2.3 cm^–1^ only, even though σ^inh^ spans from 53 to 247 cm^–1^ (chloroform) and from 123 to 463 cm^–1^ (acetonitrile). It should be highlighted that the high accuracy of predictions of σ^inh^ based on the employed ML model results in vibronic spectra (see Figure S35 in the SI file) indistinguishable from that presented in Figure 5. We can thus conclude that the proposed machine learning model used for estimating the excitation energies for snapshots from rigid-body MD is not only very efficient but also very accurate and therefore may be used to predict the inhomogeneous broadening.

**Table 1:** Summary of Machine-Learning-Based (ML) Predictions of the Standard Deviation (σ^inh^) Corresponding to the Distribution of the Vertical Excitation Energies to the S_1_ Electronic State^a^

				σ^inh^ [cm^–1^]	
structure	σ^KRR^	MAE [cm^–1^]	MAPE, %	TD-DFT	ML
	chloroform				
B-1	0.4	13.35	0.05	56.13	58.28
B-2	0.7	15.05	0.06	136.84	137.90
B-3	0.3	20.77	0.08	121.91	121.48
B-4	0.4	14.35	0.05	161.95	163.09
B-5	0.5	12.96	0.05	247.31	247.17
	acetonitrile				
B-1	0.9	41.42	0.14	123.20	128.98
B-2	0.6	31.84	0.12	267.77	269.79
B-3	0.4	42.82	0.16	246.49	252.12
B-4	0.5	34.92	0.13	302.71	304.20
B-5	0.6	34.72	0.12	462.82	459.82

a The TD-DFT value was predicted based on 10,000 snapshots, while predictions were made based on 9,800 snapshots. σ^KRR^ is the standard deviation used in the KRR model.

## 4 Summary and Outlook

The results of electronic-structure simulations have become an invaluable support for interpreting experimental absorption/emission spectra. Nevertheless, despite continuous theory developments, the calculations of vertical excitation energies at the Franck–Condon point employing highly accurate electronic-structure theories, e.g., CC3 or CASPT2, remain impractical for most compounds of experimental interest. The palette of available electronic-structure approaches for the simulation of the vibrational fine structure of absorption/emission bands is further largely reduced since such simulations require at least one Hessian and one gradient calculation for the ground and excited states, respectively. This is why TD-DFT remains the most widely used scheme in studies of absorption/emission spectra, vibronic shapes being modeled using a specific vibrational space. Unfortunately, the successes of these vibronic approaches are inseparably connected not only to the harmonic character of the considered potential energy surfaces but also to the accuracy of the DFA used in the underlying TD-DFT calculations.

In this work, we assessed the adequacy of the VG and AH models in predicting the absorption band shapes for a set of fluorescent dyes using experimental results as benchmarks. We carefully selected adequate DFAs and used several schemes to account for environmental effects. The selection of DFAs was achieved using the vibrational reorganization energy as a handy metric and taking gas-phase RI-CC2 values as references. This procedure revealed that the spread in λ_vib_ is large across the tested DFAs (from hundreds to thousands cm^–1^) and that the most accurate results are obtained with ωB97X, LC-BLYP, and its optimally tuned variant LC-BLYP-OT(α)–all three delivering average relative errors smaller than 20%. This preselection approach appeared robust as it led to satisfying band shapes with respect to the reference approach; yet, more sophisticated methods than RI-CC2 might be employed in further works, provided one is looking for ultimate benchmarks. The selected ωB97X and LC-BLYP-OT(α) DFAs, delivering alike band shapes in the gas phase, were next used in more sophisticated calculations of the vibrational fine structure of the absorption bands in solution. To this end, both the VG and AH vibronic approaches were used and combined with the PCM model. The comparison with measured bands shapes revealed the very valuable performance of the VG scheme, despite the large structural variability in the 20-dye set. In contrast, the performance of the AH model was comparatively poor with too long (and in some cases unphysical) vibrational progressions in many cases. One can partially alleviate the flaws of the AH model by using geometries estimated from excited-state gradients and ground-state Hessian in the FC region. In short, by and large, the simple and robust VG model proved to be, in all considered cases, accurate in predicting the band shapes; this success partially originates in the rational preselection of DFAs.

An important ingredient of bandwidth simulations is the inhomogeneous broadening induced by the solvent microenvironment. The line shape function corresponding to this inhomogeneous broadening, after convolution with stick spectra representing Franck–Condon transitions, allows for nonempirical determination of absorption band shapes. In the present study, we made an effort to assess the performance of the electrostatic embedding approach combined with the selected DFAs for predicting the inhomogeneous broadening based on solute–solvent configurations obtained from rigid-body MD simulations. It turned out that with combining the preselected DFAs, the VG and EE approaches lead to satisfactory nonempirical band widths with errors in final fwhm’s not exceeeding 25%, even though the employed protocol does not account for vibrational (electrical and mechanical) anharmonicity nor self-consistent solute–solvent polarization. Given the successful applications of machine learning models in the field of optical spectroscopies,^118,119^ we have employed a kernel ridge regression machine learning model, combined with a newly proposed low-dimensional Coulomb-type fingerprint, to reduce the computational cost of inhomogeneous broadening estimations. This approach proved to be extremely robust offering inhomogeneous broadenings with errors as small as 2 cm^–1^ with respect to genuine electronic-structure calculations, with the total CPU time reduced by 98%. Given the robustness and accuracy of this approach, it can likely be extended to more expensive solvation models like polarizable embedding or to more complex environments like membranes or peptides, where the Marcus model cannot be easily applied.^14^

## Supporting Information Available

Synthesis of new dyes, experimental electronic absorption spectra, choosing DFA for the simulations of vibrationally-resolved electronic absorption spectra, and validation of VG and AH models (PDF)
