# Supplementary material for: Cost-Effective Simulations of Vibrationally-Resolved Absorption Spectra of Fluorophores with Machine-Learning-Based Inhomogeneous Broadening
Source: J Chem Theory Comput. 2023 Apr 5;19(8):2304–15. doi: 10.1021/acs.jctc.2c01285 (PMC10134414; doi:10.1021/acs.jctc.2c01285)
Supplement: Supplementary file 1 — ct2c01285_si_001.pdf [file ct2c01285_si_001.pdf]

Supporting Information: Cost-Effective Simulations of Vibrationally-Resolved Absorption Spectra of Fluorophores with Machine-Learning-Based Inhomogeneous Broadening

Elizaveta F. Petrusevich,[†,‡] Manon H. E. Bousquet,[¶] Borys Ośmiałowski,[§] Denis Jacquemin,[*,¶,‖] Josep M. Luis,[*,⊥] and Robert Zaleśny[*,†]

†*Faculty of Chemistry, Wrocław University of Science and Technology, Wyb. Wyspiańskiego 27, PL–50370 Wrocław, Poland*

‡*Institute of Computational Chemistry and Catalysis and Department of Chemistry, University of Girona, Campus de Montilivi, 17003 Girona, Catalonia, Spain*

¶*Nantes Université, CNRS, CEISAM UMR 6230, F-44000 Nantes, France*

§*Faculty of Chemistry, Nicolaus Copernicus University, Gagarina Street 7, PL-87-100 Toruń, Poland*

‖*Institut Universitaire de France (IUF), F-75005, Paris, France*

⊥*Institute of Computational Chemistry and Catalysis and Department of Chemistry, University of Girona, Campus de Montilivi, 17071 Girona, Catalonia, Spain*

E-mail: Denis.Jacquemin@univ-nantes.fr; Josepm.Luis@udg.edu; Robert.Zalesny@pwr.edu.pl

Contents

Experimental Spectra S-3

Choosing DFA for the Simulations of Vibrationally-Resolved Electronic Absorption Spectra S-8

Validation of VG and AH Models S-18

References **S-45**

Experimental Spectra

The set of compounds consists of several structures. Whole series **B**[S1,S2] and **C**[S3] were described before. The same is realized for structures **A-3**,[S4] **A-7**,[S5] **A-8** and **A-9**.[S6]

For the compounds listed below the following new syntheses were guided.

A-1. The synthesis of β-diketone was guided as before,[S7] while difluoroborate was obtained as other BF_2 carrying diketonates.[S8]

A-2. The 2(benzoylmethylene)quinoline was synthesized as described in publication of one of authors,[S9] while its complexation with BF_3 etherate as described elsewhere.[S10]

A-4. The compound was obtained in two step reaction. First the 2,6-diamino(pyridin-2-yl)pyridine was obtained[S11] and, after purification, it was proceeded as in another reference.[S12]

A-5, **A-6**. Both compounds were obtained using known procedure for the synthesis of unsubstituted derivative[S5] but two other esters were used in the synthesis of amides, namely, ethyl 4-metoxybenzoate and methyl 4-trifluoromethylbenzoate in the reaction. Substitution of acidic proton was guided as usual, using BF_3 etherate in presence of DIEA in DCM as a solvent.

A detailed synthetic procedures and photophysical studies will be published in a separate article. The absorption spectra were recorded using Shimadzu UV-1900 spectrometer in 1 cm qartz cells.

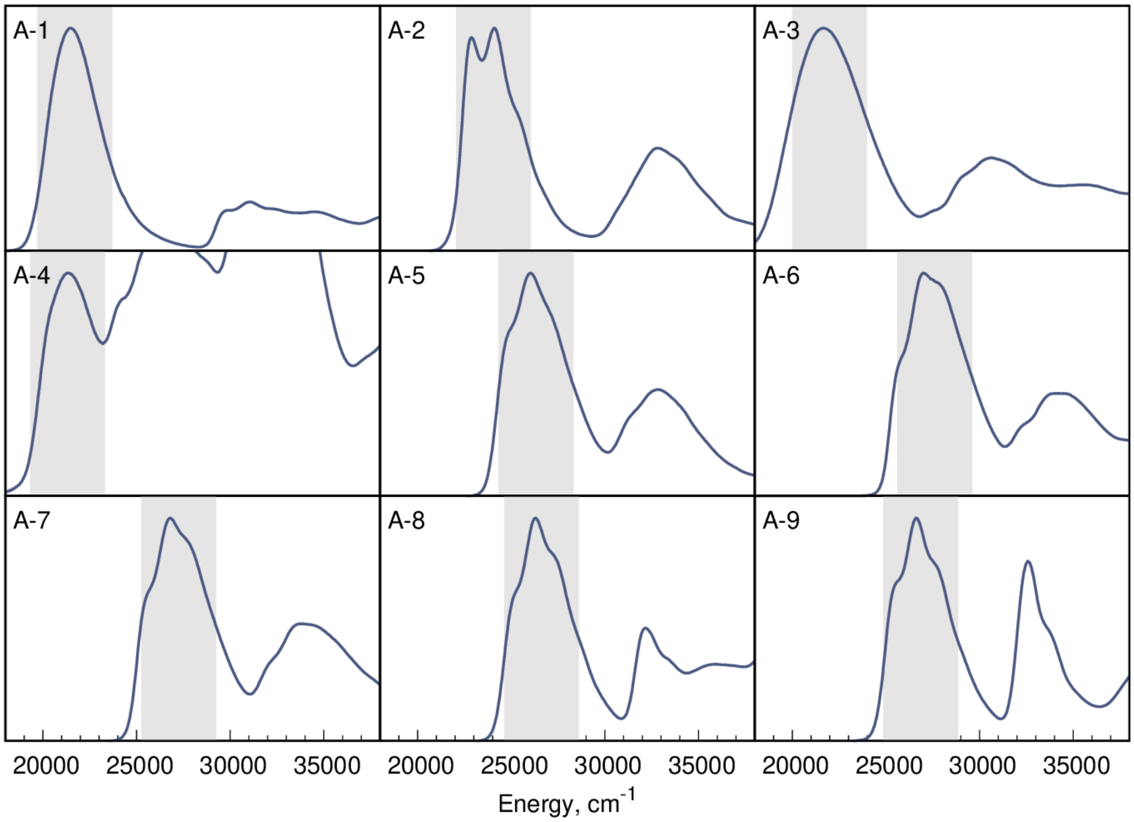

Figure S1: Normalized absorption spectra of studied dyes from set A in chloroform solvent. The gray vertical bar (width = 4000 cm^{-1}) is the guide to the eye.

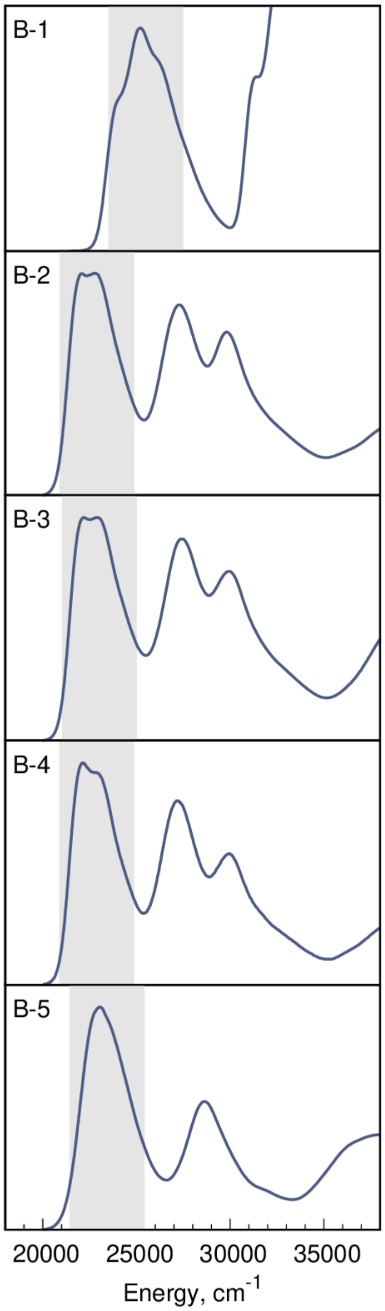

Figure S2: Normalized absorption spectra of studied dyes from set B in chloroform solvent. The gray vertical bar (width = 4000 cm^{-1}) is the guide to the eye.

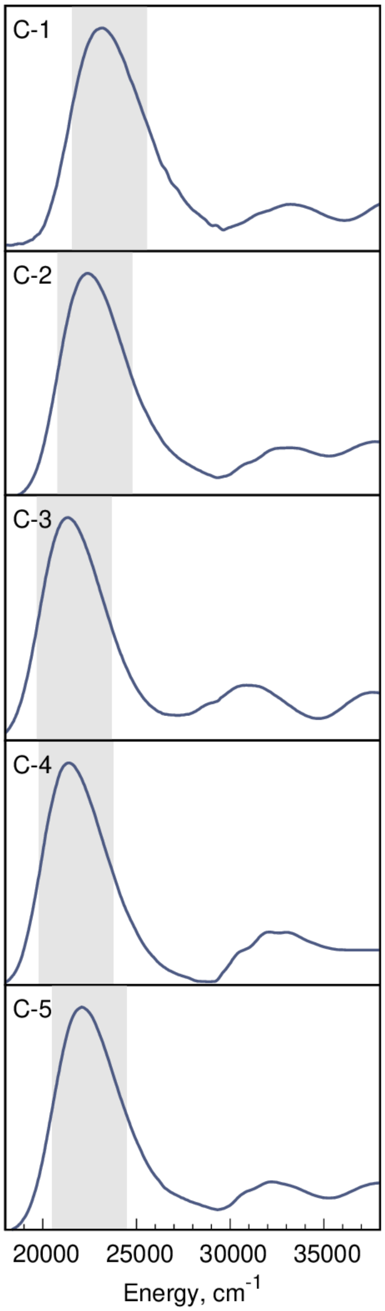

Figure S3: Normalized absorption spectra of studied dyes from set C in chloroform solvent. The gray vertical bar (width = 4000 cm^{-1}) is the guide to the eye.

Table S1: Full width at half maximum corresponding to spectra shown in Figs. S1-S3.

Structure	FWHM, cm^{-1}
**A-1**	3095
**A-2**	3452
**A-3**	4685
**A-4**	2990
**A-5**	3938
**A-6**	4228
**A-7**	4136
**A-8**	3595
**A-9**	3590
**B-1**	3888
**B-2**	3173
**B-3**	3324
**B-4**	3022
**B-5**	3008
**C-1**	4608
**C-2**	4172
**C-3**	4011
**C-4**	3950
**C-5**	4102

Choosing DFA for the Simulations of Vibrationally-Resolved Electronic Absorption Spectra

Table S2: Vibrational reorganization energies achieved for studied compounds within 12 DFAs and CC2 in the gas phase

	B3LYP	BLYP	LC-BLYP	LC-BLYP-OT(J)	LC-BLYP-OT(α)	PBE0	ωB97XD	BH&HLYP	CAM-B3LYP	M06-2X	MN15	ωB97X	CC2
	**Vibrational reorganization energy, cm^{-1}**												
**A-1**	1965.28	2773.58	1567.92	847.10	1136.77	1769.59	1251.72	1348.35	1157.66	1263.53	1156.30	1432.57	1744.76
**A-2**	1117.62	2257.84	2064.17	1132.40	1485.06	1141.28	1526.13	1609.57	1503.91	1512.84	1253.03	1759.12	1608.09
**A-3**	453.23	1304.00	3122.65	1583.12	2431.17	570.64	2377.86	2062.94	2117.75	2159.90	1304.86	2924.17	2720.26
**A-4**	1722.10	3131.52	1330.19	1062.86	1132.94	1594.86	1136.96	1297.62	1176.06	1218.95	1195.29	1200.62	1434.43
**A-5**	1093.15	2872.88	2096.72	1388.96	1663.88	1007.71	1542.94	1524.77	1476.84	1599.83	1270.49	1823.43	1949.04
**A-6**	1513.26	1046.58	2339.55	1740.96	1979.71	1625.91	1914.70	2125.70	1950.31	2161.06	1885.67	2098.35	2450.47
**A-7**	1211.75	1416.09	2246.00	1635.34	1847.97	1326.56	1771.94	1871.58	1773.16	1943.77	1657.05	1990.56	2315.87
**A-8**	1475.45	1180.66	2301.60	1550.54	1829.04	1539.75	1795.79	2062.62	1849.18	1931.76	1728.20	2006.56	2007.66
**A-9**	1302.52	843.91	2336.68	1527.44	1812.24	1372.41	1765.61	2013.15	1808.33	1859.25	1648.89	2008.75	1968.36
**B-1**	1509.97	1549.92	2187.27	1571.25	1896.97	1631.36	1856.87	1977.97	1878.49	2006.50	1766.71	2024.20	2209.60
**B-2**	749.27	392.21	1788.77	1243.80	1506.54	848.20	1444.40	1416.46	1373.53	1392.83	1185.93	1676.94	1730.10
**B-3**	3968.71	3179.60	1796.47	1231.75	1524.65	4076.64	1481.39	1505.87	1463.71	1434.00	1198.17	1686.22	1763.16
**B-4**	650.65	362.83	1629.62	1059.78	1346.31	740.23	1310.84	1287.31	1226.62	1227.40	1050.66	1524.23	1578.90
**B-5**	2648.44	4314.32	1852.91	1229.79	1542.54	1969.14	1433.03	1459.28	1351.91	1320.28	1193.24	1673.98	1788.08
**C-1**	565.88	2324.86	2660.78	1240.73	1937.13	500.51	2016.28	1681.27	1590.84	1612.47	945.87	2581.31	2413.42
**C-2**	1033.62	4899.33	2759.57	1076.15	1860.63	607.01	1571.24	1513.89	1245.57	1165.19	768.90	2636.22	2363.20
**C-3**	1431.49	3583.89	2382.05	985.69	1657.28	1003.26	1372.68	1373.92	1124.56	1085.53	766.08	2240.38	2122.60
**C-4**	537.48	2232.57	2613.36	980.00	1732.81	435.79	1396.94	1341.48	1114.27	1045.23	685.58	2549.90	2186.76
**C-5**	2263.23	4402.22	2554.62	980.69	1707.13	1401.91	1421.74	1426.53	1125.04	1073.27	732.24	2812.13	2233.46
**C-6**	2263.52	2557.53	4503.91	2124.22	3202.51	1690.40	2917.91	2186.20	2241.14	2949.62	1559.13	4077.52	4014.21
	**Relative errors wrt CC2, %**												
	B3LYP	BLYP	LC-BLYP	LC-BLYP-OT(J)	LC-BLYP-OT(α)	PBE0	ωB97XD	BH&HLYP	CAM-B3LYP	M06-2X	MN15	ωB97X	
**A-1**	12.64	58.97	10.14	51.45	34.85	1.42	28.26	22.72	33.65	27.58	33.73	17.89	
**A-2**	30.50	40.41	28.36	29.58	7.65	29.03	5.10	0.09	6.48	5.92	22.08	9.39	
**A-3**	83.34	52.06	14.79	41.80	10.63	79.02	12.59	24.16	22.15	20.60	52.03	7.50	
**A-4**	20.05	118.31	7.27	25.90	21.02	11.18	20.74	9.54	18.01	15.02	16.67	16.30	
**A-5**	43.91	47.40	7.58	28.74	14.63	48.30	20.84	21.77	24.23	17.92	34.81	6.44	
**A-6**	38.25	57.29	4.53	28.95	19.21	33.65	21.86	13.25	20.41	11.81	23.05	14.37	
**A-7**	47.68	38.85	3.02	29.39	20.20	42.72	23.49	19.18	23.43	16.07	28.45	14.05	
**A-8**	26.51	41.19	14.64	22.77	8.90	23.31	10.55	2.74	7.89	3.78	13.92	0.05	
**A-9**	33.83	57.13	18.71	22.40	7.93	30.28	10.30	2.28	8.13	5.54	16.23	2.05	
**B-1**	31.66	29.86	1.01	28.89	14.15	26.17	15.96	10.48	14.99	9.19	20.04	8.39	
**B-2**	56.69	77.33	3.39	28.11	12.92	50.97	16.51	18.13	20.61	19.49	31.45	3.07	
**B-3**	125.09	80.34	1.89	30.14	13.53	131.21	15.98	14.59	16.98	18.67	32.04	4.36	
**B-4**	58.79	77.02	3.21	32.88	14.73	53.12	16.98	18.47	22.31	22.26	33.46	3.46	
**B-5**	48.12	141.28	3.63	31.22	13.73	10.13	19.86	18.39	24.39	26.16	33.27	6.38	
**C-1**	76.55	3.67	10.25	48.59	19.74	79.26	16.46	30.34	34.08	33.19	60.81	6.96	
**C-2**	56.26	107.32	16.77	54.46	21.27	74.31	33.51	35.94	47.29	50.69	67.46	11.55	
**C-3**	32.56	68.84	12.22	53.56	21.92	52.73	35.33	35.27	47.02	48.86	63.91	5.55	
**C-4**	75.42	2.09	19.51	55.18	20.76	80.07	36.12	38.65	49.04	52.20	68.65	16.61	
**C-5**	1.33	97.10	14.38	56.09	23.57	37.23	36.34	36.13	49.63	51.95	67.21	25.91	
**C-6**	43.61	36.29	12.20	47.08	20.22	57.89	27.31	45.54	44.17	26.52	61.16	1.58	
	**Average relative errors wrt CC2, %**												
	47.14	61.64	10.37	37.36	17.08	47.60	21.20	20.88	26.74	24.17	39.02	9.09	

Table S3: Signed absolute and relative errors in vibrational reorganization energies for studied compounds within 12 DFAs wrt. CC2 in the gas phase

Vibrational reorganization energy: Signed absolute errors wrt. CC2, cm^{-1}

	B3LYP	BLYP	LC-BLYP	LC-BLYP-OT(J)	LC-BLYP-OT(α)	PBE0	ωB97XD	BH&HLYP	CAM-B3LYP	M06-2X	MN15	ωB97X
**A-1**	220.52	1028.82	-176.84	-897.66	-607.99	24.83	-493.04	-396.41	-587.10	-481.23	-588.46	-312.19
**A-2**	-490.47	649.75	456.08	-475.69	-123.03	-466.81	-81.96	1.48	-104.18	-95.25	-355.06	151.03
**A-3**	-2267.03	-1416.26	402.39	-1137.14	-289.09	-2149.62	-342.40	-657.32	-602.51	-560.36	-1415.40	203.91
**A-4**	287.67	1697.09	-104.24	-371.57	-301.49	160.43	-297.47	-136.81	-258.37	-215.48	-239.14	-233.81
**A-5**	-855.89	923.84	147.68	-560.08	-285.16	-941.33	-406.10	-424.27	-472.20	-349.21	-678.55	-125.61
**A-6**	-937.21	-1403.89	-110.92	-709.51	-470.76	-824.56	-535.77	-324.77	-500.16	-289.41	-564.80	-352.12
**A-7**	-1104.12	-899.78	-69.87	-680.53	-467.90	-989.31	-543.93	-444.29	-542.71	-372.10	-658.82	-325.31
**A-8**	-532.21	-827.00	293.94	-457.12	-178.62	-467.91	-211.87	54.96	-158.48	-75.90	-279.46	-1.10
**A-9**	-665.84	-1124.45	368.32	-440.92	-156.12	-595.95	-202.75	44.79	-160.03	-109.11	-319.47	40.39
**B-1**	-699.63	-659.68	-22.33	-638.35	-312.63	-578.24	-352.73	-231.63	-331.11	-203.10	-442.89	-185.40
**B-2**	-980.83	-1337.89	58.67	-486.30	-223.56	-881.90	-285.70	-313.64	-356.57	-337.27	-544.17	-53.16
**B-3**	2205.55	1416.44	33.31	-531.41	-238.51	2313.48	-281.77	-257.29	-299.45	-329.16	-564.99	-76.94
**B-4**	-928.25	-1216.07	50.72	-519.12	-232.59	-838.67	-268.06	-291.59	-352.28	-351.50	-528.24	-54.67
**B-5**	860.36	2526.24	64.83	-558.29	-245.54	181.06	-355.05	-328.80	-436.17	-467.80	-594.84	-114.10
**C-1**	-1847.54	-88.56	247.36	-1172.69	-476.29	-1912.91	-397.14	-732.15	-822.58	-800.95	-1467.55	167.89
**C-2**	-1329.58	2536.13	396.37	-1287.05	-502.57	-1756.19	-791.96	-849.31	-1117.63	-1198.01	-1594.30	273.02
**C-3**	-691.11	1461.29	259.45	-1136.91	-465.32	-1119.34	-749.92	-748.68	-998.04	-1037.07	-1356.52	117.78
**C-4**	-1649.28	45.81	426.60	-1206.76	-453.95	-1750.97	-789.82	-845.28	-1072.49	-1141.53	-1501.18	363.14
**C-5**	29.77	2168.76	321.16	-1252.77	-526.33	-831.55	-811.72	-806.93	-1108.42	-1160.19	-1501.22	578.67
**C-6**	-1750.69	-1456.68	489.70	-1889.99	-811.70	-2323.81	-1096.30	-1828.01	-1773.07	-1064.59	-2455.08	63.31

Signed relative errors wrt CC2, %

	B3LYP	BLYP	LC-BLYP	LC-BLYP-OT(J)	LC-BLYP-OT(α)	PBE0	ωB97XD	BH&HLYP	CAM-B3LYP	M06-2X	MN15	ωB97X
**A-1**	12.64	58.97	-10.14	-51.45	-34.85	1.42	-28.26	-22.72	-33.65	-27.58	-33.73	-17.89
**A-2**	-30.50	40.41	28.36	-29.58	-7.65	-29.03	-5.10	0.09	-6.48	-5.92	-22.08	9.39
**A-3**	-83.34	-52.06	14.79	-41.80	-10.63	-79.02	-12.59	-24.16	-22.15	-20.60	-52.03	7.50
**A-4**	20.05	118.31	-7.27	-25.90	-21.02	11.18	-20.74	-9.54	-18.01	-15.02	-16.67	-16.30
**A-5**	-43.91	47.40	7.58	-28.74	-14.63	-48.30	-20.84	-21.77	-24.23	-17.92	-34.81	-6.44
**A-6**	-38.25	-57.29	-4.53	-28.95	-19.21	-33.65	-21.86	-13.25	-20.41	-11.81	-23.05	-14.37
**A-7**	-47.68	-38.85	-3.02	-29.39	-20.20	-42.72	-23.49	-19.18	-23.43	-16.07	-28.45	-14.05
**A-8**	-26.51	-41.19	14.64	-22.77	-8.90	-23.31	-10.55	2.74	-7.89	-3.78	-13.92	-0.05
**A-9**	-33.83	-57.13	18.71	-22.40	-7.93	-30.28	-10.30	2.28	-8.13	-5.54	-16.23	2.05
**B-1**	-31.66	-29.86	-1.01	-28.89	-14.15	-26.17	-15.96	-10.48	-14.99	-9.19	-20.04	-8.39
**B-2**	-56.69	-77.33	3.39	-28.11	-12.92	-50.97	-16.51	-18.13	-20.61	-19.49	-31.45	-3.07
**B-3**	125.09	80.34	1.89	-30.14	-13.53	131.21	-15.98	-14.59	-16.98	-18.67	-32.04	-4.36
**B-4**	-58.79	-77.02	3.21	-32.88	-14.73	-53.12	-16.98	-18.47	-22.31	-22.26	-33.46	-3.46
**B-5**	48.12	141.28	3.63	-31.22	-13.73	10.13	-19.86	-18.39	-24.39	-26.16	-33.27	-6.38
**C-1**	-76.55	-3.67	10.25	-48.59	-19.74	-79.26	-16.46	-30.34	-34.08	-33.19	-60.81	6.96
**C-2**	-56.26	107.32	16.77	-54.46	-21.27	-74.31	-33.51	-35.94	-47.29	-50.69	-67.46	11.55
**C-3**	-32.56	68.84	12.22	-53.56	-21.92	-52.73	-35.33	-35.27	-47.02	-48.86	-63.91	5.55
**C-4**	-75.42	2.09	19.51	-55.18	-20.76	-80.07	-36.12	-38.65	-49.04	-52.20	-68.65	16.61
**C-5**	1.33	97.10	14.38	-56.09	-23.57	-37.23	-36.34	-36.13	-49.63	-51.95	-67.21	25.91
**C-6**	-43.61	-36.29	12.20	-47.08	-20.22	-57.89	-27.31	-45.54	-44.17	-26.52	-61.16	1.58

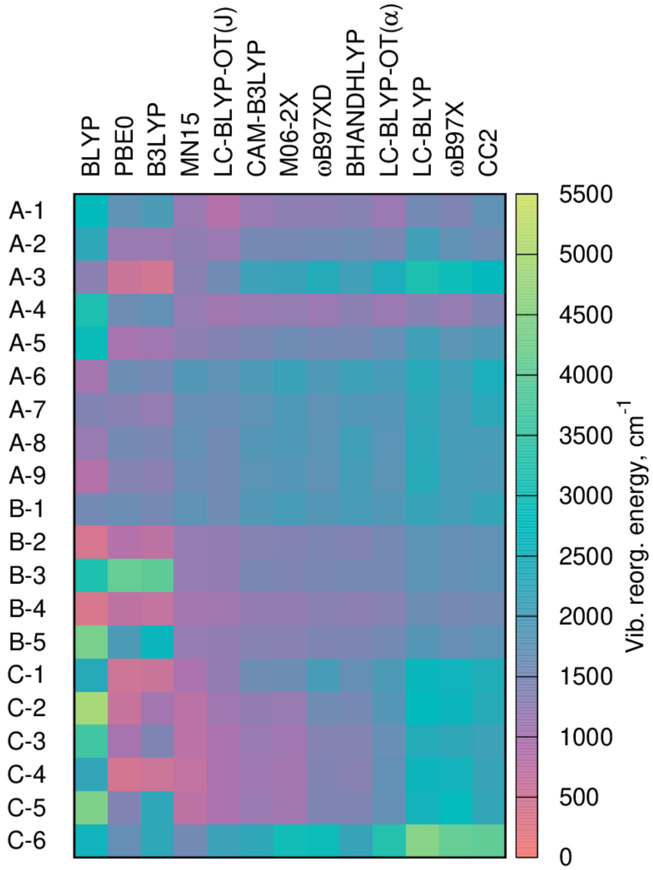

Figure S4: The plot demonstrating the range of vibrational reorganization energies achieved for studied compounds within 12 DFAs and CC2 in the gas phase

Table S4: Vertical excitation energies achieved for studied compounds within 12 DFAs and CC2 in the gas phase

	B3LYP	BLYP	LC-BLYP	LC-BLYP-OT(J)	LC-BLYP-OT(α)	PBE0	ωB97XD	BH&HLYP	CAM-B3LYP	M06-2X	MN15	ωB97X	CC2
**Vertical excitation energy, eV ($S_0 \rightarrow S_1$ transition)**													
**A-1**	24335.92	19737.65	31634.60	27348.47	29264.08	25443.35	29294.73	29537.51	28700.29	28243.77	27042.78	30448.13	26014.64
**A-2**	24792.44	20271.60	30650.58	25986.98	28132.46	25691.77	28225.22	28897.09	27992.12	27929.20	26699.18	29296.34	26069.00
**A-3**	22458.22	18563.28	30552.99	25291.71	27934.85	23420.46	27325.89	27302.50	26757.25	26349.13	24996.50	28980.97	25425.92
**A-4**	22300.94	18305.17	27271.04	23847.94	25376.40	23348.68	25503.03	26241.05	25242.51	25200.57	24137.50	26287.83	22748.83
**A-5**	29438.30	24064.11	35861.04	32080.63	33416.32	30719.14	33517.95	34389.05	33238.07	33391.32	31812.85	34572.14	31494.42
**A-6**	31638.63	27964.69	36916.04	33163.06	34718.13	32686.37	34651.18	35673.92	34520.52	34852.02	33297.75	35600.52	32943.34
**A-7**	31494.26	27570.28	36761.98	33145.31	34420.50	32585.55	34521.32	35541.64	34392.27	34668.93	33159.02	35460.98	32737.18
**A-8**	27478.33	23974.58	33936.56	29361.68	31228.09	28497.84	31015.15	31780.59	30850.61	30900.62	29485.08	31015.15	29086.80
**A-9**	27855.81	24456.91	34183.37	29785.93	31507.97	28824.50	31265.19	32049.18	31133.72	31178.89	29773.03	32546.83	29592.03
**B-1**	25569.98	21053.17	31821.72	26783.06	29404.43	26687.08	29476.21	30175.51	29065.66	29470.56	27921.94	30540.08	27155.22
**B-2**	23547.90	18716.53	30473.94	25989.40	27950.17	24694.04	28033.25	28640.60	27588.02	27571.89	26108.77	29242.30	25411.00
**B-3**	20716.83	13866.60	30637.68	25901.48	28159.08	22563.88	28239.73	28723.68	27660.61	27795.31	26325.74	29409.26	25594.09
**B-4**	23810.84	18883.49	30703.81	26022.46	28199.41	24977.95	28288.93	28932.58	27901.78	27842.09	26377.36	29471.37	25845.18
**B-5**	23222.04	16940.45	31434.57	26737.89	29027.76	24736.79	29001.95	29506.86	28526.87	28420.41	26846.78	30169.06	26353.80
**C-1**	24031.84	19397.27	31922.55	27183.93	29349.58	25124.75	29002.75	29185.84	28448.64	28035.67	26744.35	30498.14	26788.39
**C-2**	23555.96	17884.95	31710.42	26849.20	29041.47	24754.53	28743.84	28951.13	28138.91	27646.90	26421.72	30279.56	26226.69
**C-3**	22389.66	17152.58	30857.06	26189.43	28353.46	23620.49	27954.21	28015.51	27350.08	26966.96	26421.72	29417.33	25404.71
**C-4**	23642.27	19473.90	31307.13	26599.16	28721.26	24672.26	28413.95	28598.66	27828.38	27331.53	26157.16	29870.62	25685.96
**C-5**	22617.11	16313.75	31755.59	26854.85	29089.06	24068.94	28747.07	28786.59	28109.07	27634.80	26282.18	30261.01	26256.53
**C-6**	22351.75	18503.59	31041.77	25185.24	27762.24	23293.02	27448.49	27689.65	26865.33	26118.45	24706.14	29412.49	24787.68
**Relative errors wrt CC2, %**													
	B3LYP	BLYP	LC-BLYP	LC-BLYP-OT(J)	LC-BLYP-OT(α)	PBE0	ωB97XD	BH&HLYP	CAM-B3LYP	M06-2X	MN15	ωB97X	
**A-1**	6.5	24.1	21.6	5.1	12.5	2.2	12.6	13.5	10.3	8.6	4.0	17.0	
**A-2**	4.9	22.2	17.6	0.3	7.9	1.4	8.3	10.8	7.4	7.1	2.4	12.4	
**A-3**	11.7	27.0	20.2	0.5	9.9	7.9	7.5	7.4	5.2	3.6	1.7	14.0	
**A-4**	2.0	19.5	19.9	4.8	11.6	2.6	12.1	15.4	11.0	10.8	6.1	15.6	
**A-5**	6.5	23.6	13.9	1.9	6.1	2.5	6.4	9.2	5.5	6.0	1.0	9.8	
**A-6**	4.0	15.1	12.1	0.7	5.4	0.8	5.2	8.3	4.8	5.8	1.1	8.1	
**A-7**	3.8	15.8	12.3	1.2	5.1	0.5	5.4	8.6	5.1	5.9	1.3	8.3	
**A-8**	5.5	17.6	16.7	0.9	7.4	2.0	6.6	9.3	6.1	6.2	1.4	6.6	
**A-9**	5.9	17.4	15.5	0.7	6.5	2.6	5.7	8.3	5.2	5.4	0.6	10.0	
**B-1**	5.8	22.5	17.2	1.4	8.3	1.7	8.5	11.1	7.0	8.5	2.8	12.5	
**B-2**	7.3	26.3	19.9	2.3	10.0	2.8	10.3	12.7	8.6	8.5	2.7	15.1	
**B-3**	19.1	45.8	19.7	1.2	10.0	11.8	10.3	12.2	8.1	8.6	2.9	14.9	
**B-4**	7.9	26.9	18.8	0.7	9.1	3.4	9.5	11.9	8.0	7.7	2.1	14.0	
**B-5**	11.9	35.7	19.3	1.5	10.1	6.1	10.0	12.0	8.2	7.8	1.9	14.5	
**C-1**	10.3	27.6	19.2	1.5	9.6	6.2	8.3	8.9	6.2	4.7	0.2	13.8	
**C-2**	10.2	31.8	20.9	2.4	10.7	5.6	9.6	10.4	7.3	5.4	0.7	15.5	
**C-3**	11.9	32.5	21.5	3.1	11.6	7.0	10.0	10.3	7.7	6.1	4.0	15.8	
**C-4**	8.0	24.2	21.9	3.6	11.8	3.9	10.6	11.3	8.3	6.4	1.8	16.3	
**C-5**	13.9	37.9	20.9	2.3	10.8	8.3	9.5	9.6	7.1	5.2	0.1	15.3	
**C-6**	9.8	25.4	25.2	1.6	12.0	6.0	10.7	11.7	8.4	5.4	0.3	18.7	
**Average relative errors wrt CC2, %**													
	8.3	25.9	18.7	1.9	9.3	4.3	8.8	10.6	7.3	6.7	2.0	13.4	

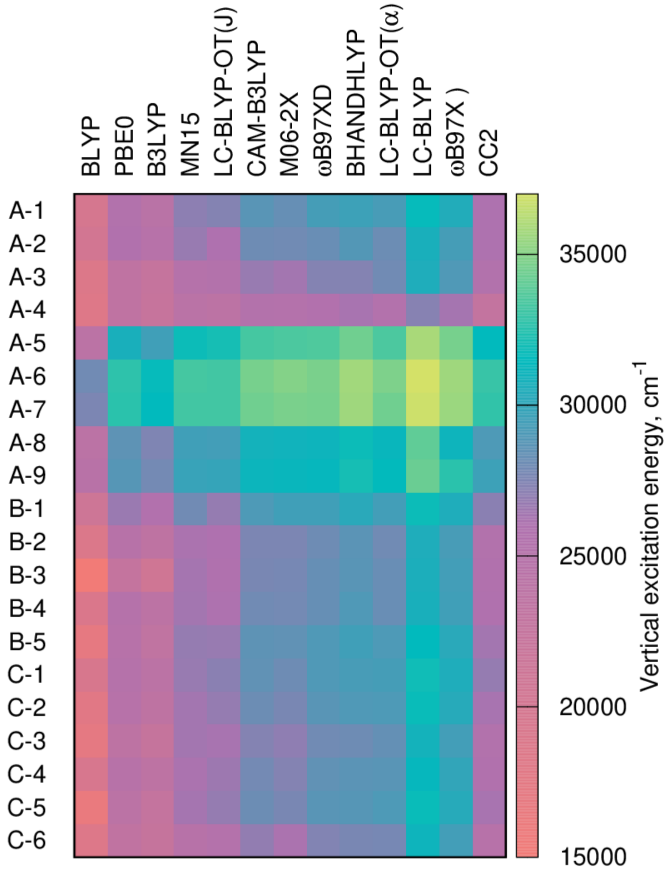

Figure S5: The plot demonstrating the range of vertical excitation energies achieved for studied compounds within 12 DFAs and CC2 in the gas phase

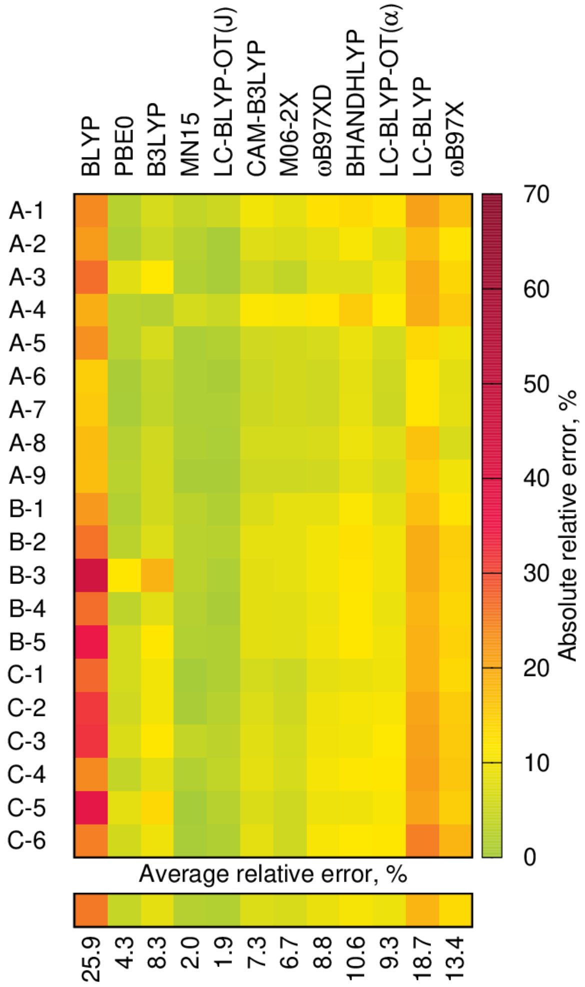

Figure S6: The plot demonstrating absolute relative errors of vertical excitation energies achieved for studied compounds within 12 DFAs wrt. CC2 in the gas phase

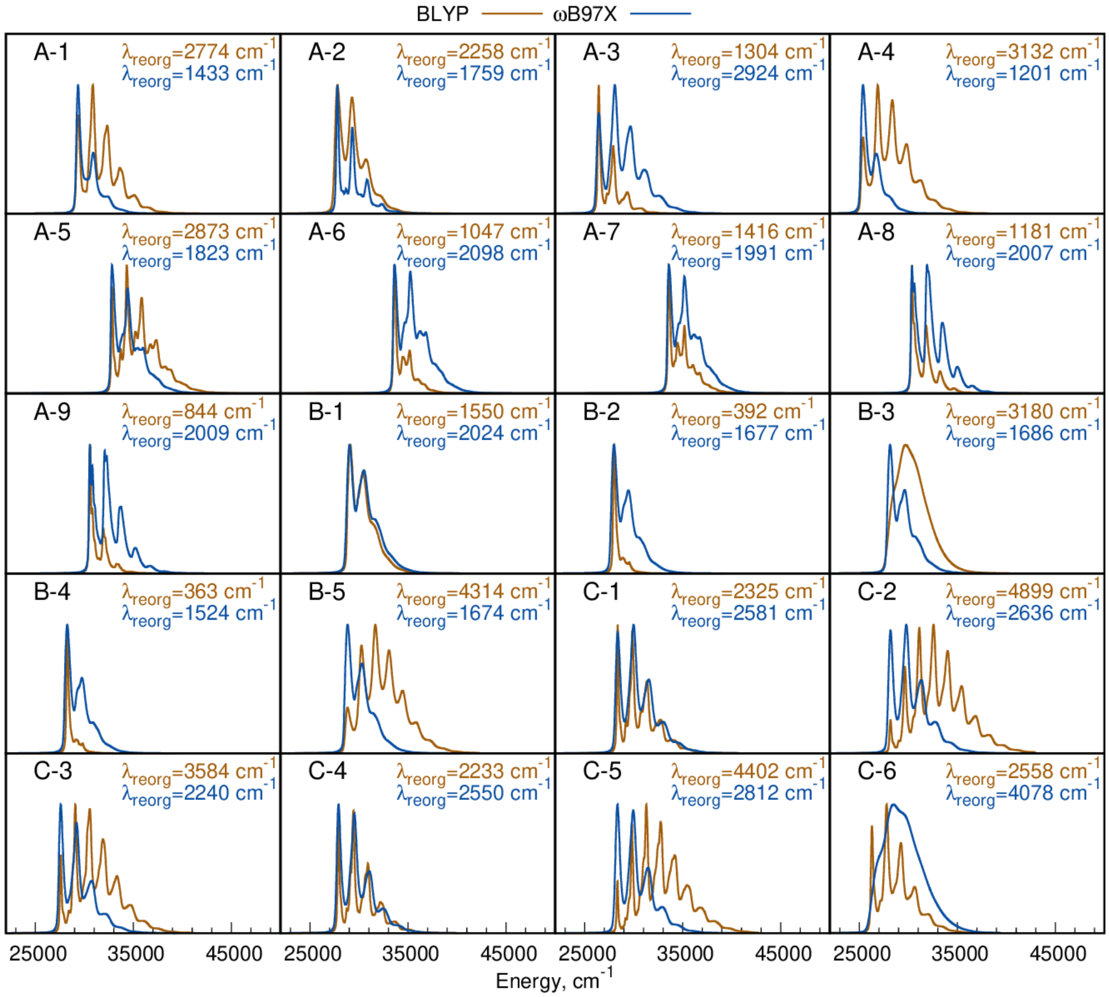

Figure S7: Comparison of vibrationally-resolved absorption spectra computed in the gas phase using ωB97X and BLYP — "the most accurate" and "the least accurate" DFAs according to evaluation method based on vibrational reorganization energy

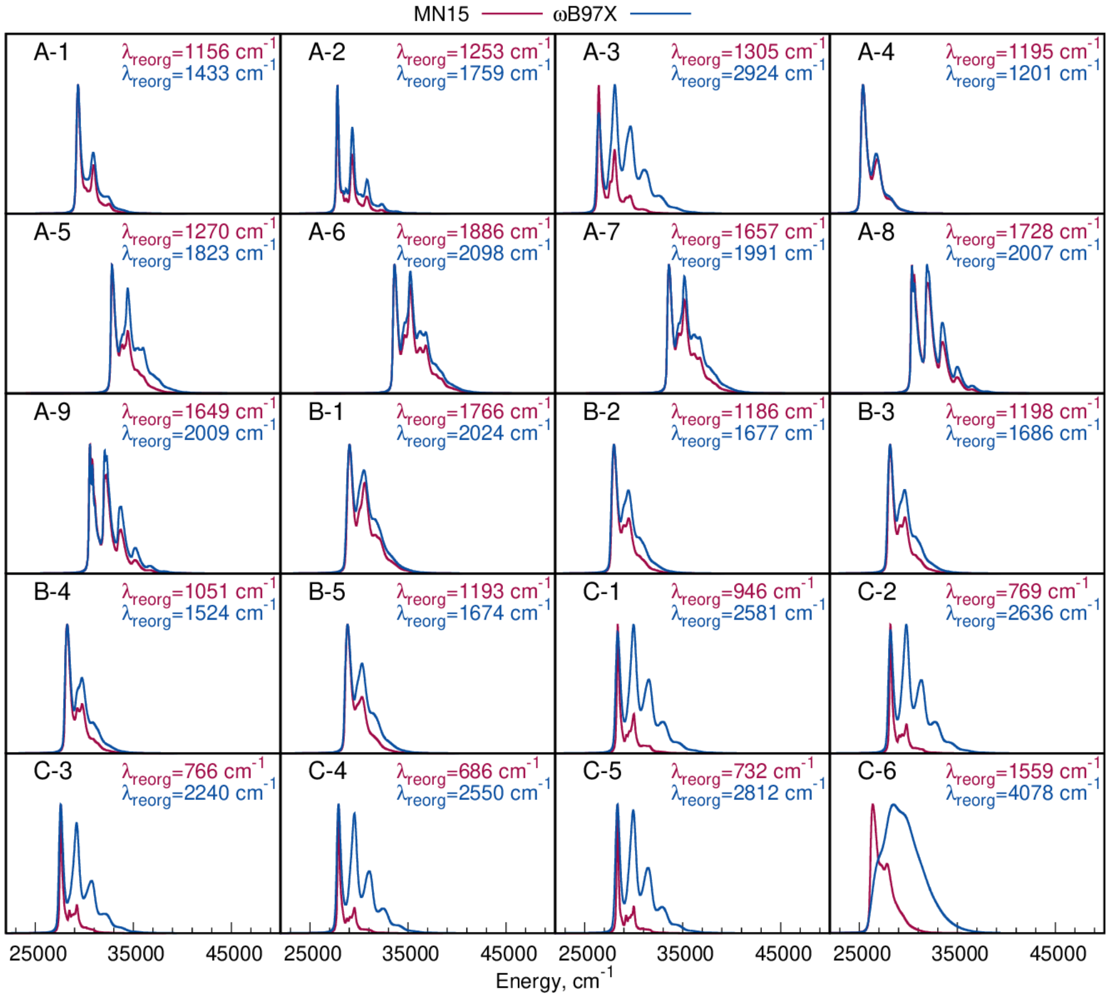

Figure S8: Comparison of vibrationally-resolved absorption spectra computed in the gas phase using ωB97X and MN15 — “the most accurate” and “mediocral” DFAs according to evaluation method based on vibrational reorganization energy

Table S5: Comparison of cc-pVDZ and aug-cc-pVDZ basis sets in calculations of vibrational reorganization energy

	**Vibrational reorganization energy, cm^{-1}**				
structure	LC-BLYP cc-pVDZ	LC-BLYP aug-cc-pVDZ	ωB97X cc-pVDZ	ωB97X aug-cc-pVDZ	CC2 cc-pVDZ
**A-1**	1567.92	1739.62	1432.57	1500.51	1744.76
**A-2**	2064.17	1787.55	1759.12	1966.01	1608.09
**A-3**	3122.65	3068.12	2924.17	2946.98	2720.26
**A-4**	1330.19	1319.85	1200.62	1185.69	1434.43
**A-5**	2096.72	2021.86	1823.43	1801.12	1949.04
**A-6**	2339.55	2247.81	2098.35	2061.85	2450.47
**A-7**	2246.00	2144.58	1990.56	1949.51	2315.87
**A-8**	2301.60	2275.26	2006.56	1997.33	2007.66
**A-9**	2336.68	2320.62	2008.75	2006.28	1968.36
**B-1**	2187.27	2137.47	2024.20	2004.56	2209.60
**B-5**	1852.91	1821.95	1673.98	1701.87	1788.08

Validation of VG and AH Models

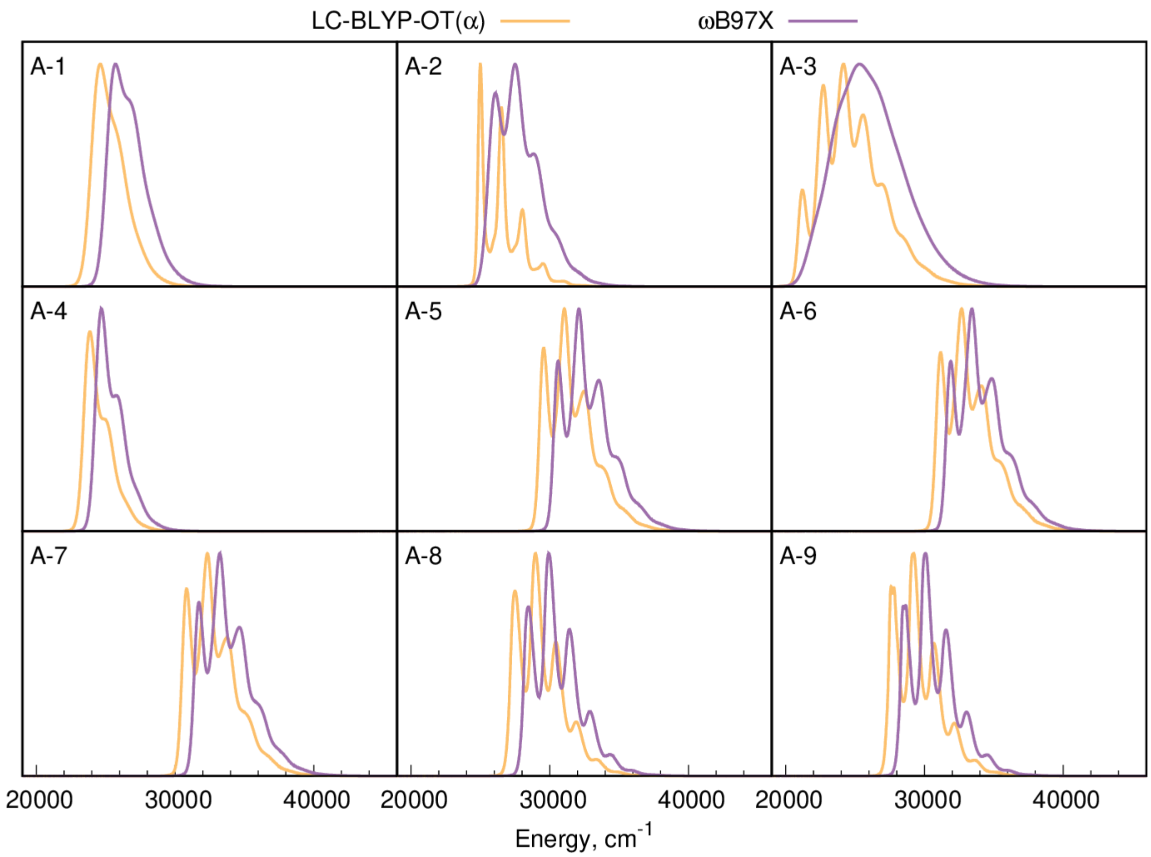

Figure S9: The comparison of two DFAs for set **A**. Shown are the spectra simulated using the VG aproach with Gaussian broadening (HWHM = 100 cm^{-1}).

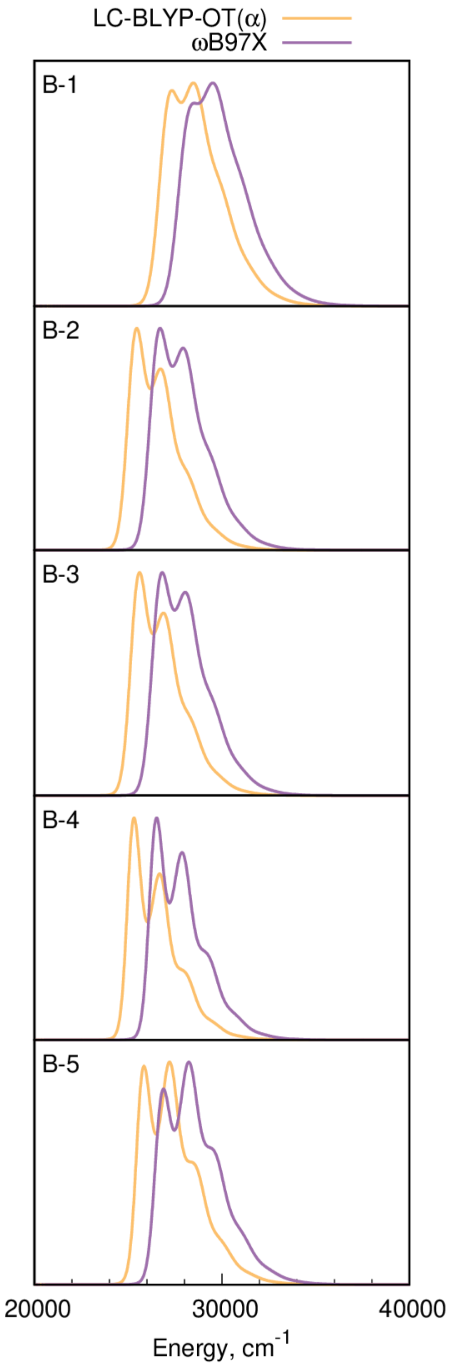

Figure S10: The comparison of two DFAs for set **B**. Shown are the spectra simulated using the VG aproach with Gaussian broadening (HWHM = 100 cm^{-1}).

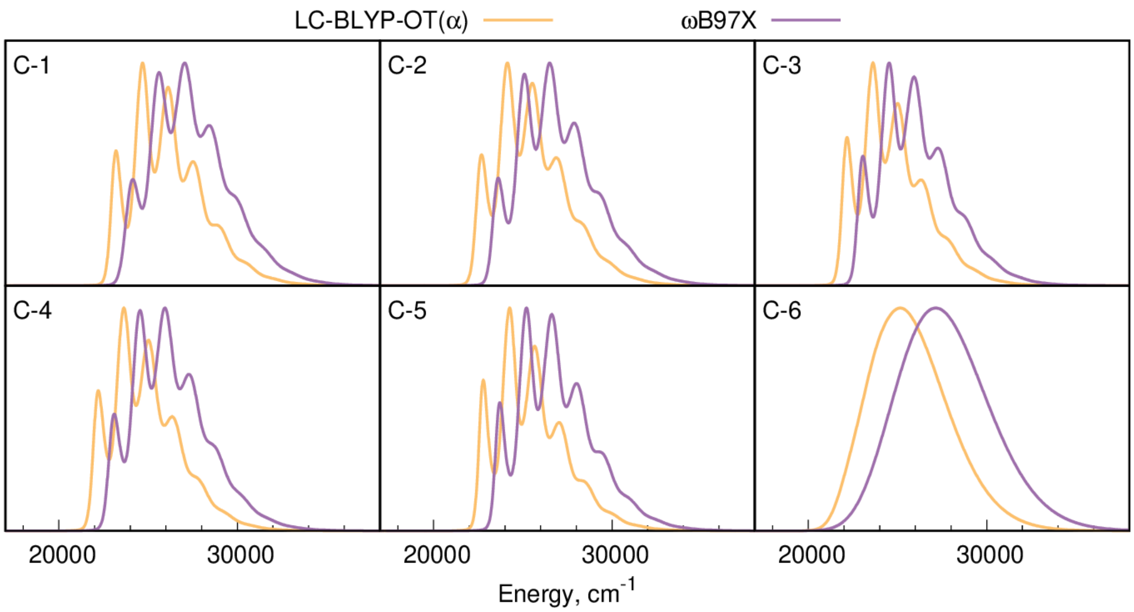

Figure S11: The comparison of two DFAs for set **C**. Shown are the spectra simulated using the VG aproach with Gaussian broadening (HWHM = 100 cm^{-1}).

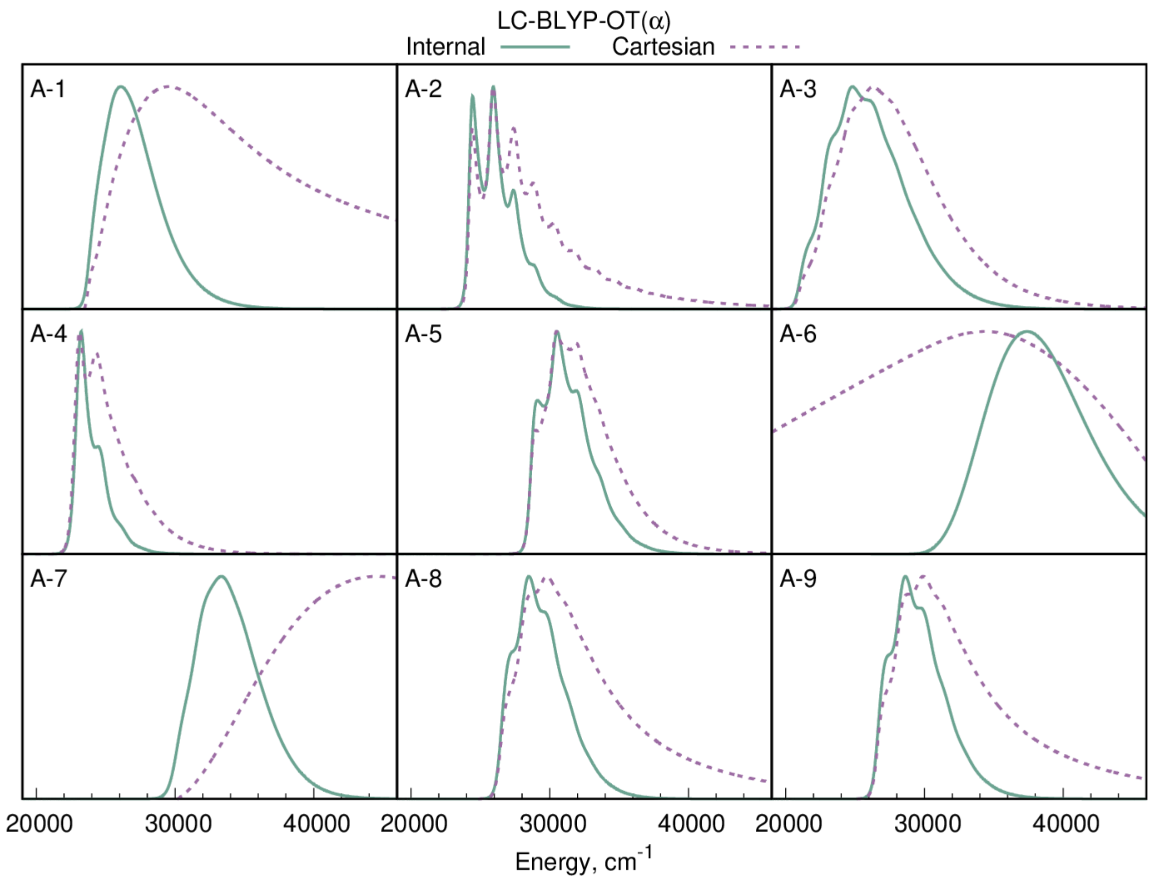

Figure S12: The comparison of Cartesian and internal coordinates calculations in AH model for set **A**. Shown are the spectra simulated using Gaussian broadening with HWHM = 100 cm^{-1}.

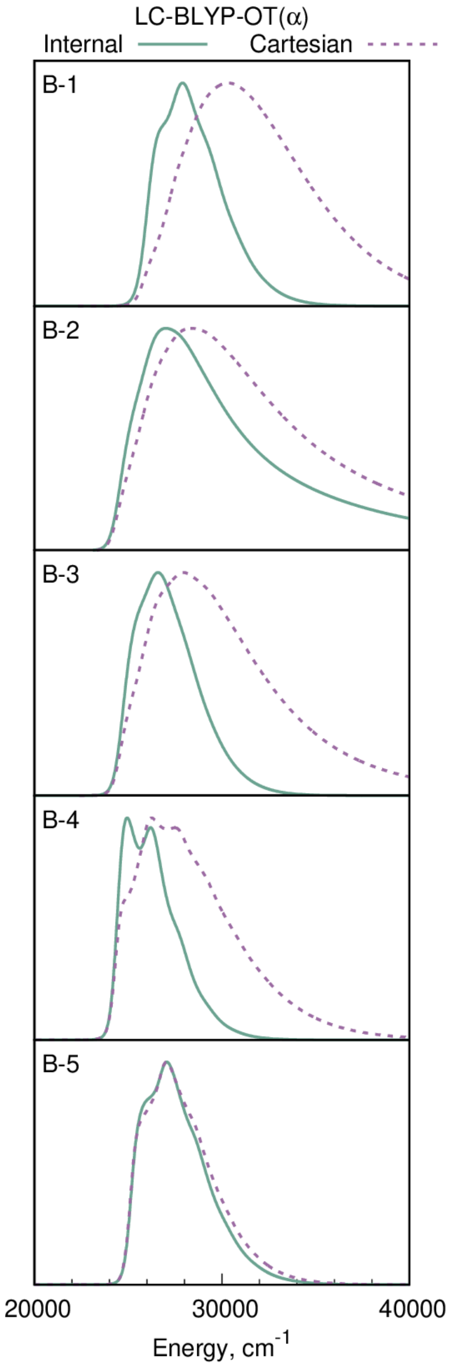

Figure S13: The comparison of Cartesian and internal coordinates calculations in AH model for set **B**. Shown are the spectra simulated using Gaussian broadening with HWHM = 100 cm^{-1}.

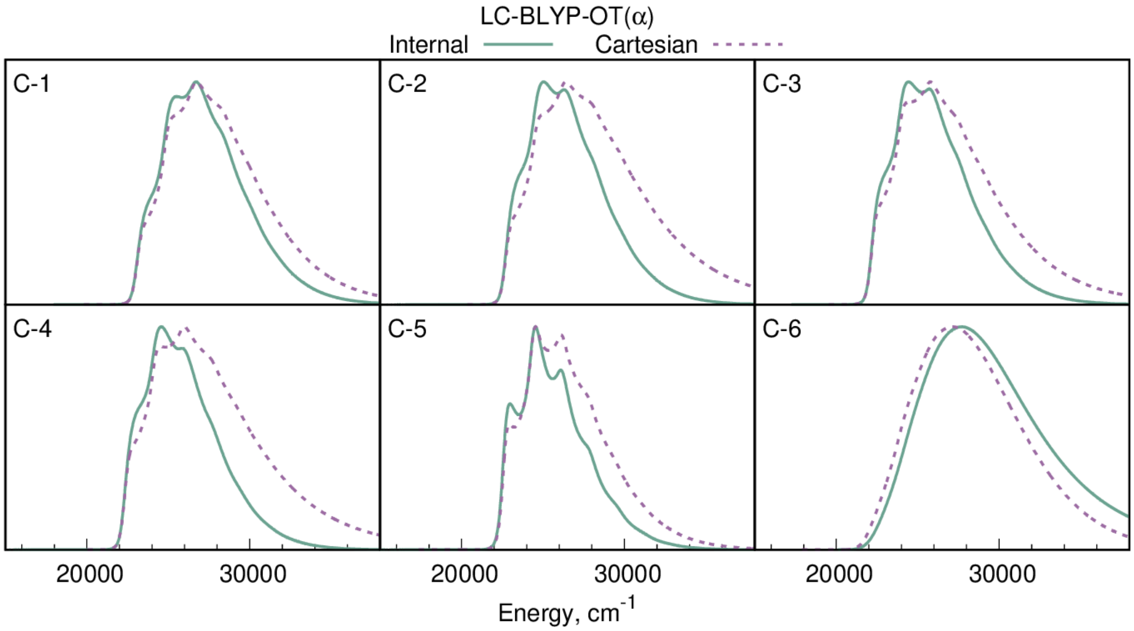

Figure S14: The comparison of Cartesian and internal coordinates calculations in AH model for set **C**. Shown are the spectra simulated using Gaussian broadening with HWHM = 100 cm^{-1}.

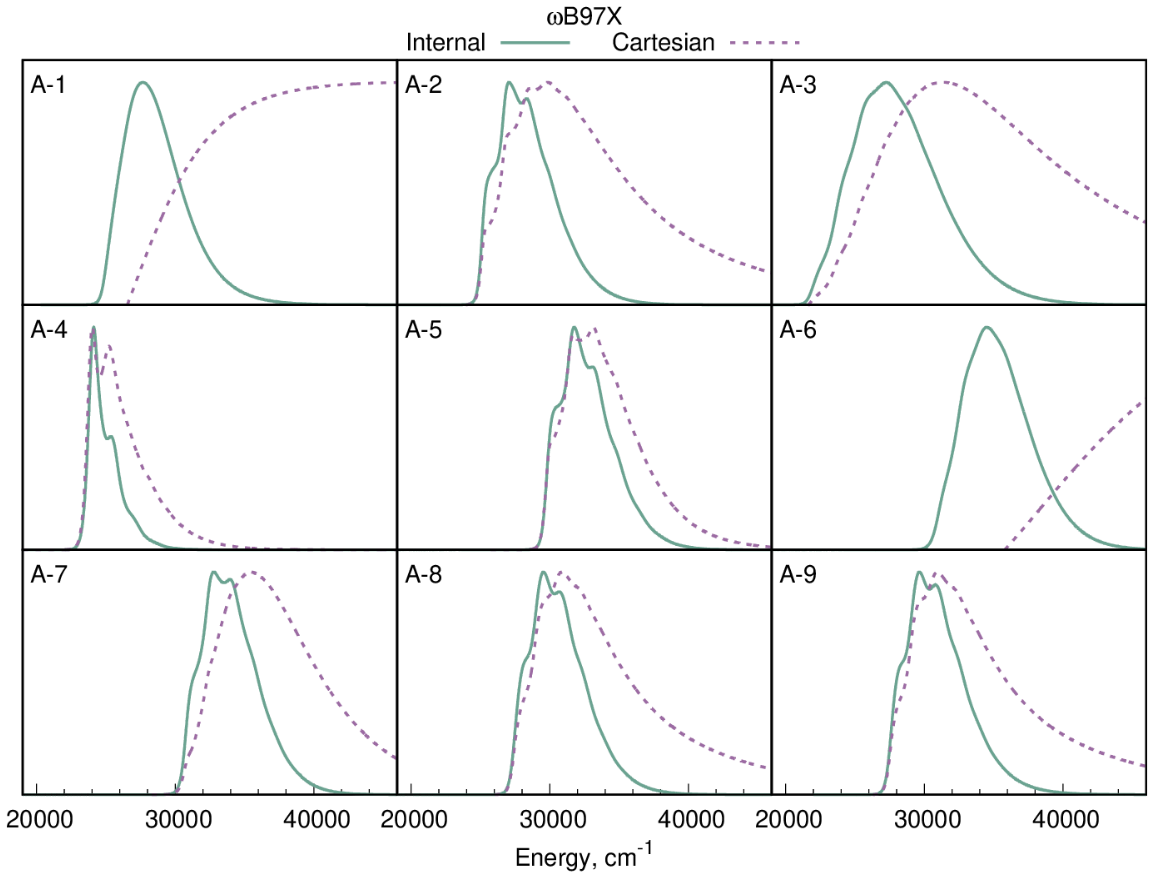

Figure S15: The comparison of Cartesian and internal coordinates calculations in AH model for set **A**. Shown are the spectra simulated using Gaussian broadening with HWHM = 100 cm^{-1}.

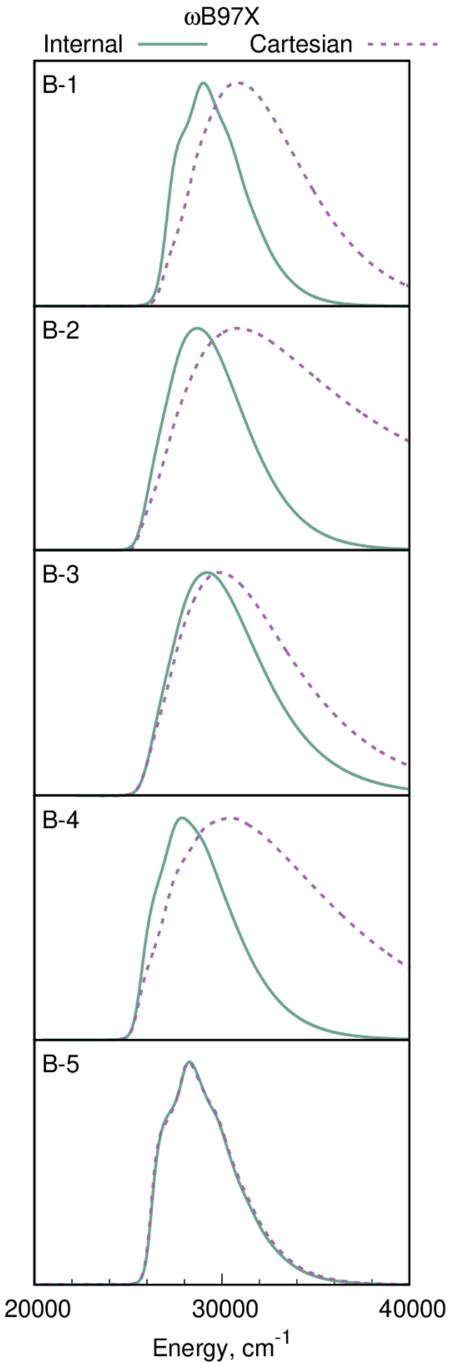

Figure S16: The comparison of Cartesian and internal coordinates calculations in AH model for set **B**. Shown are the spectra simulated using Gaussian broadening with HWHM = 100 cm^{-1}.

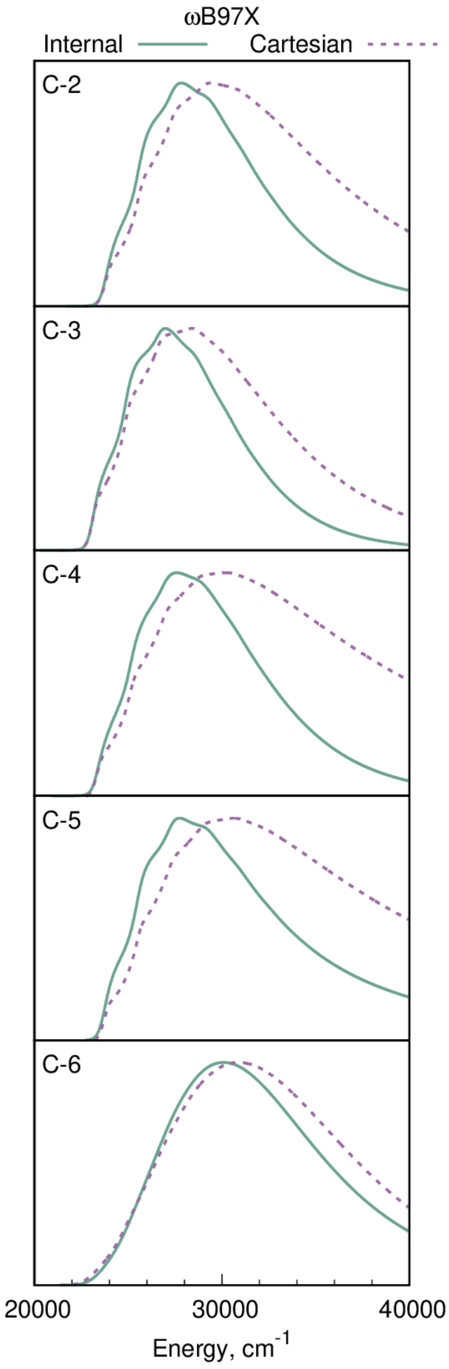

Figure S17: The comparison of Cartesian and internal coordinates calculations in AH model for set **C**. Shown are the spectra simulated using Gaussian broadening with HWHM = 100 cm^{-1}.

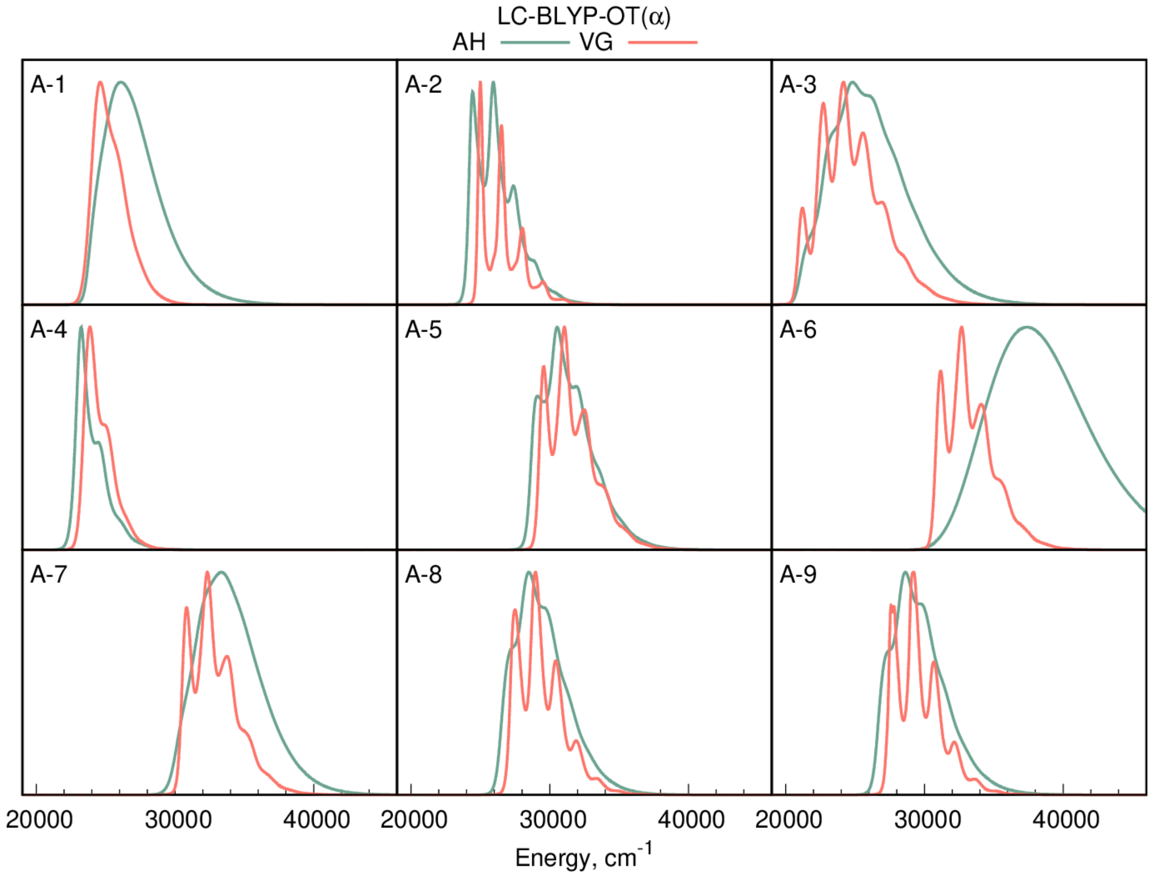

Figure S18: The comparison of the VG and AH (internal coordinates) models for series **A**. Shown are the spectra simulated using Gaussian broadening with HWHM = 100 cm^{-1}.

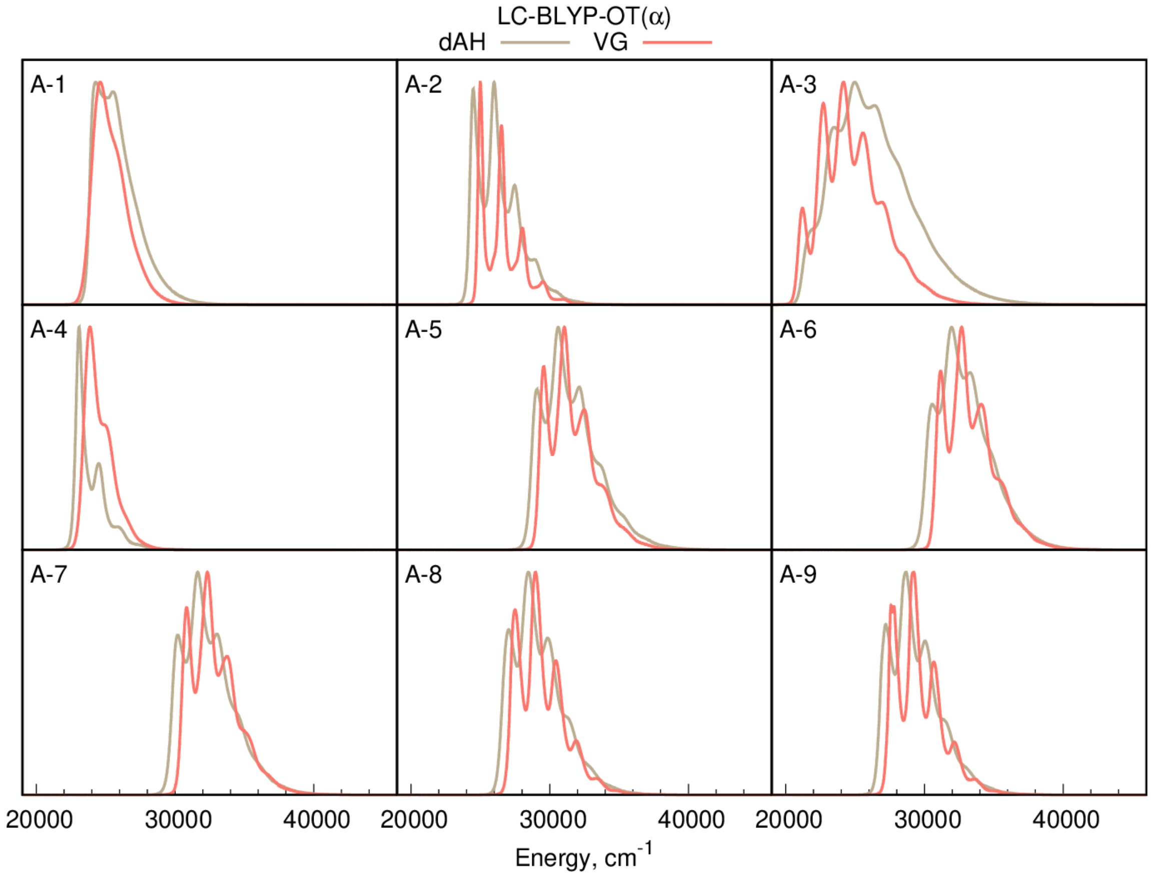

Figure S19: The comparison of the VG and dAH (internal coordinates) models for series **A**. Shown are the spectra simulated using Gaussian broadening with HWHM = 100 cm^{-1}.

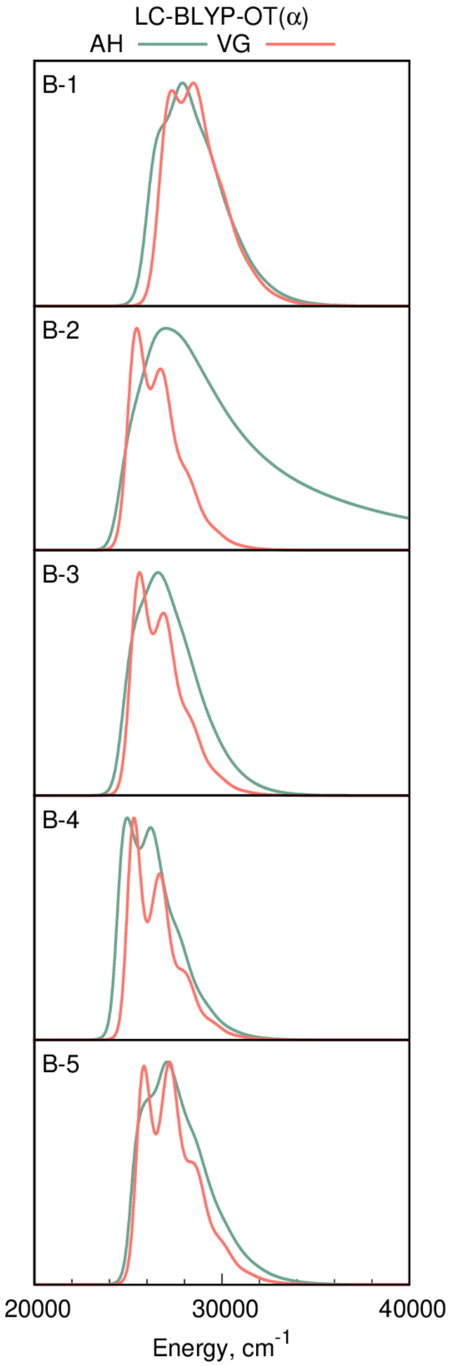

Figure S20: The comparison of the VG and AH (internal coordinates) models for series **B**. Shown are the spectra simulated using Gaussian broadening with HWHM = 100 cm^{-1}.

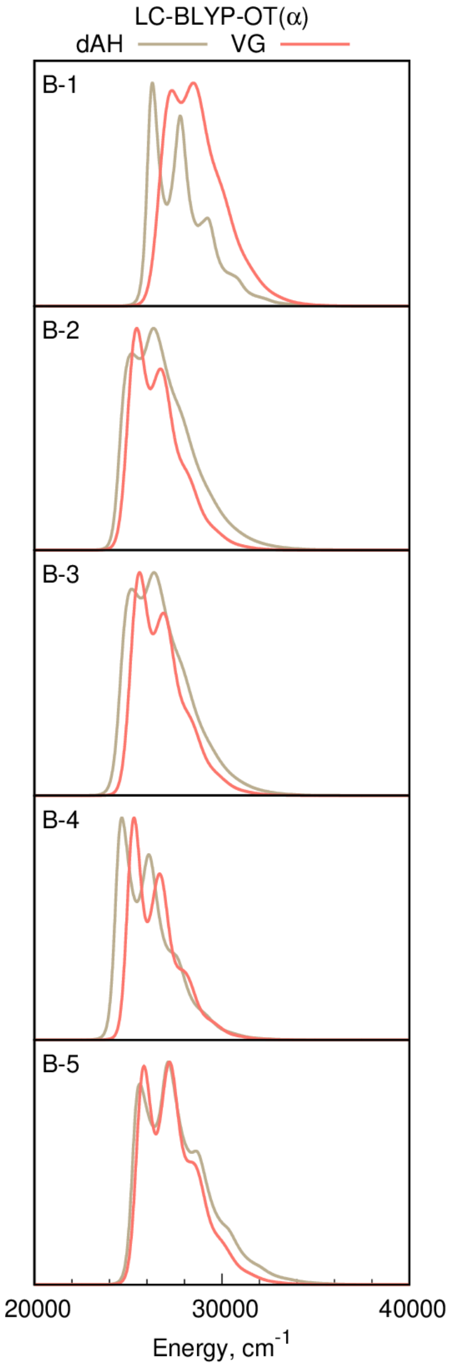

Figure S21: The comparison of the VG and dAH (internal coordinates) models for series **B**. Shown are the spectra simulated using Gaussian broadening with HWHM = 100 cm^{-1}.

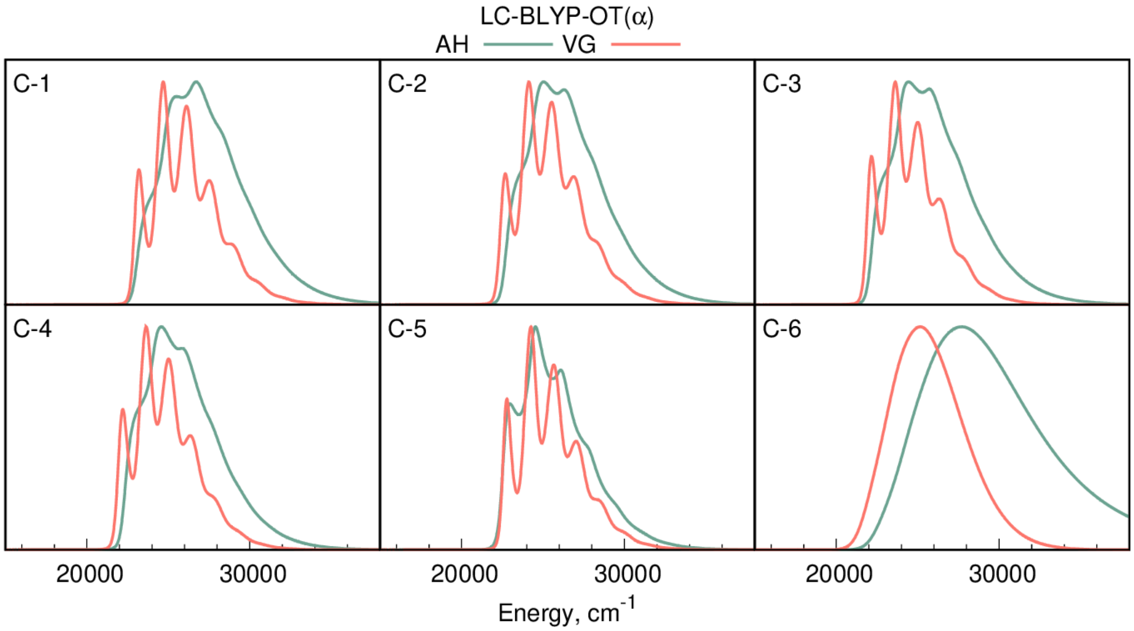

Figure S22: The comparison of the VG and AH (internal coordinates) models for series **C**. Shown are the spectra simulated using Gaussian broadening with HWHM = 100 cm^{-1}.

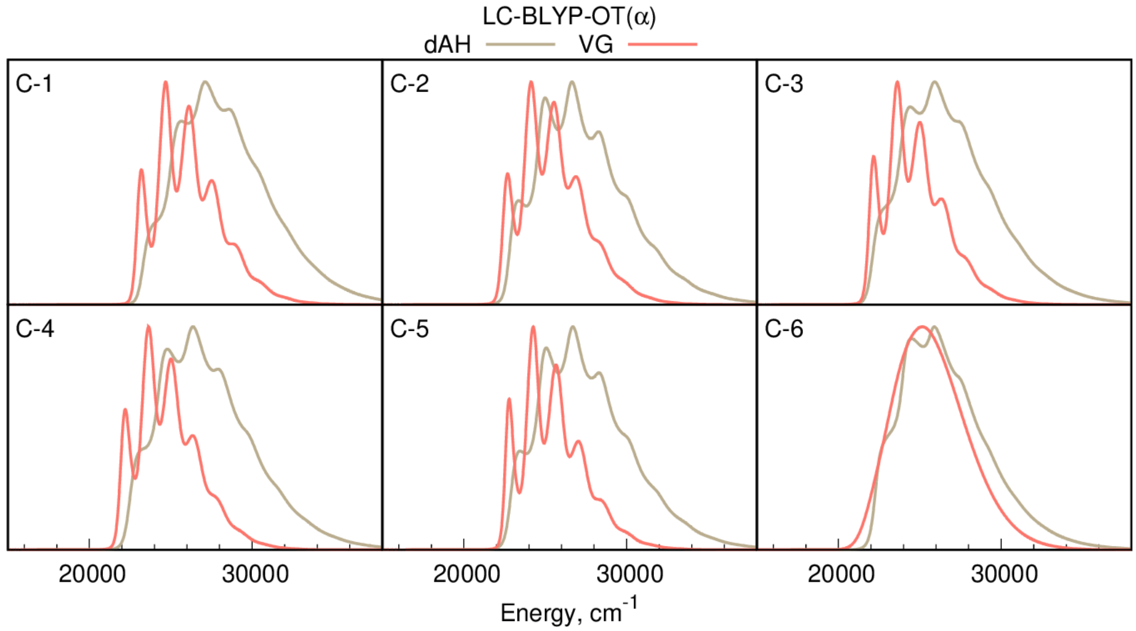

Figure S23: The comparison of the VG and dAH (internal coordinates) models for series **C**. Shown are the spectra simulated using Gaussian broadening with HWHM = 100 cm^{-1}.

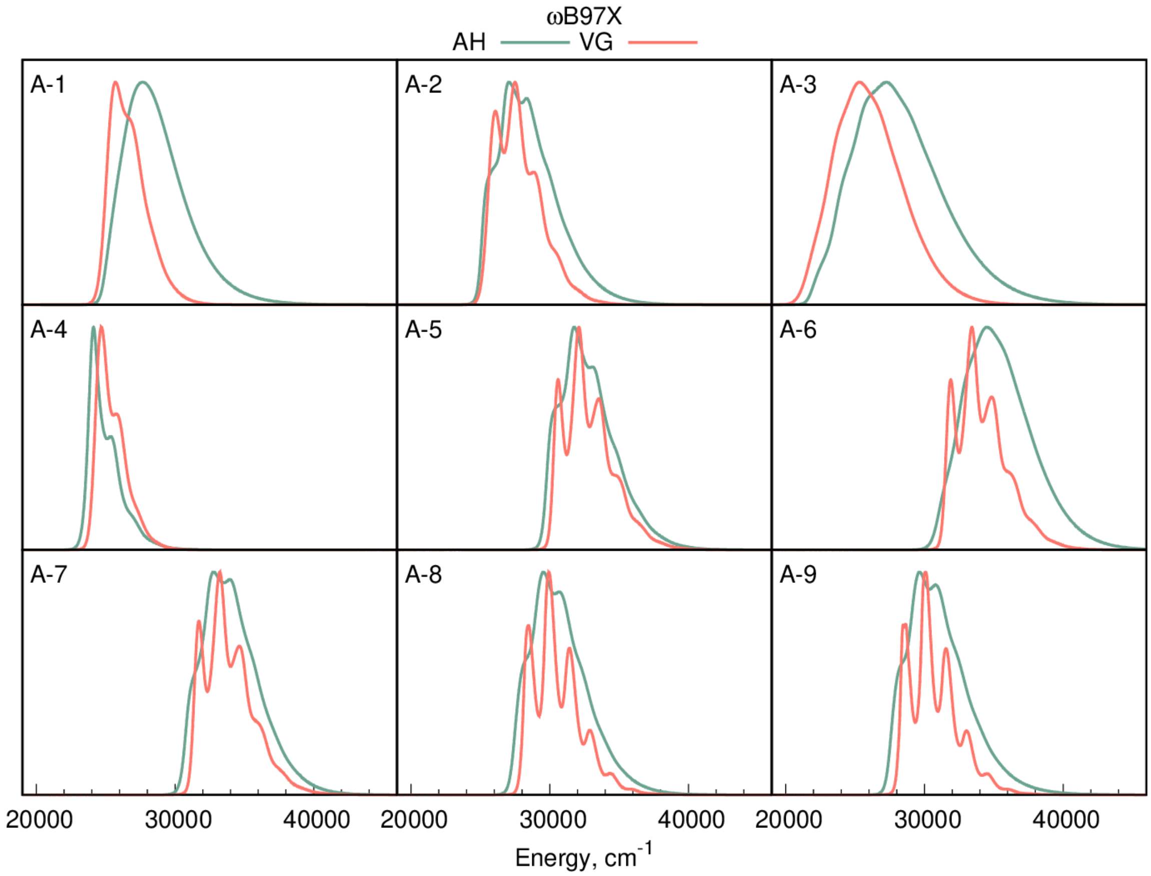

Figure S24: The comparison of the VG and AH (internal coordinates) models for series **A**. Shown are the spectra simulated using Gaussian broadening with HWHM = 100 cm^{-1}.

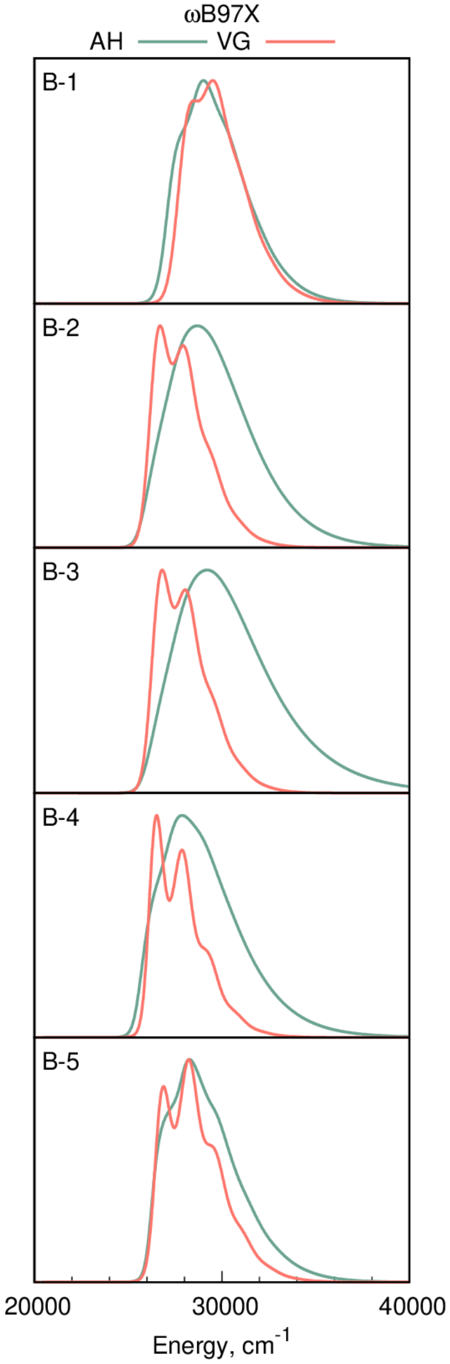

Figure S25: The comparison of the VG and AH (internal coordinates) models for series **B**. Shown are the spectra simulated using Gaussian broadening with HWHM = 100 cm^{-1}.

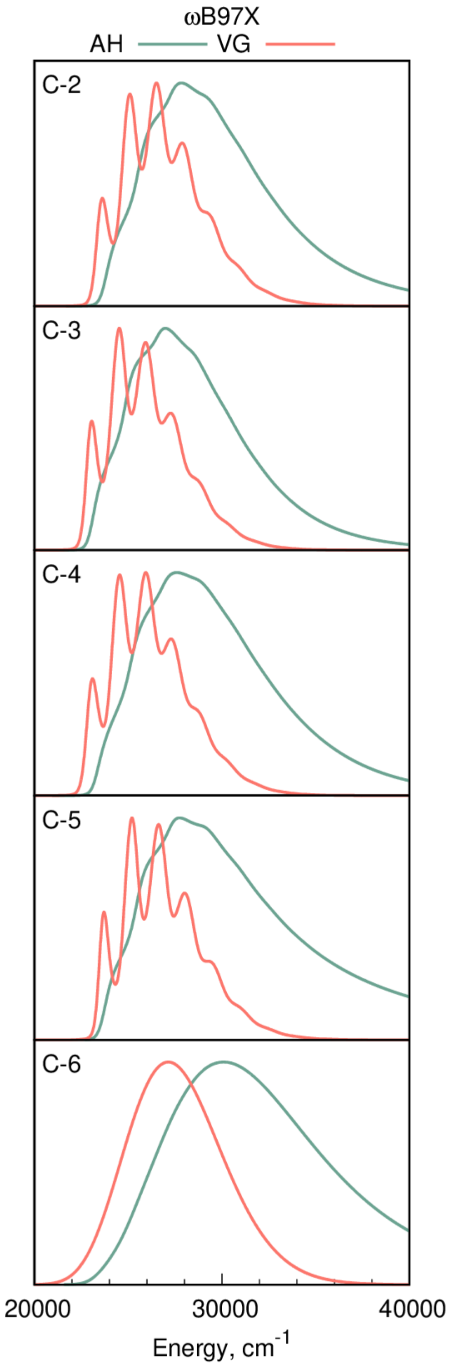

Figure S26: The comparison of the VG and AH (internal coordinates) models for series **C**. Shown are the spectra simulated using Gaussian broadening with HWHM = 100 cm^{-1}.

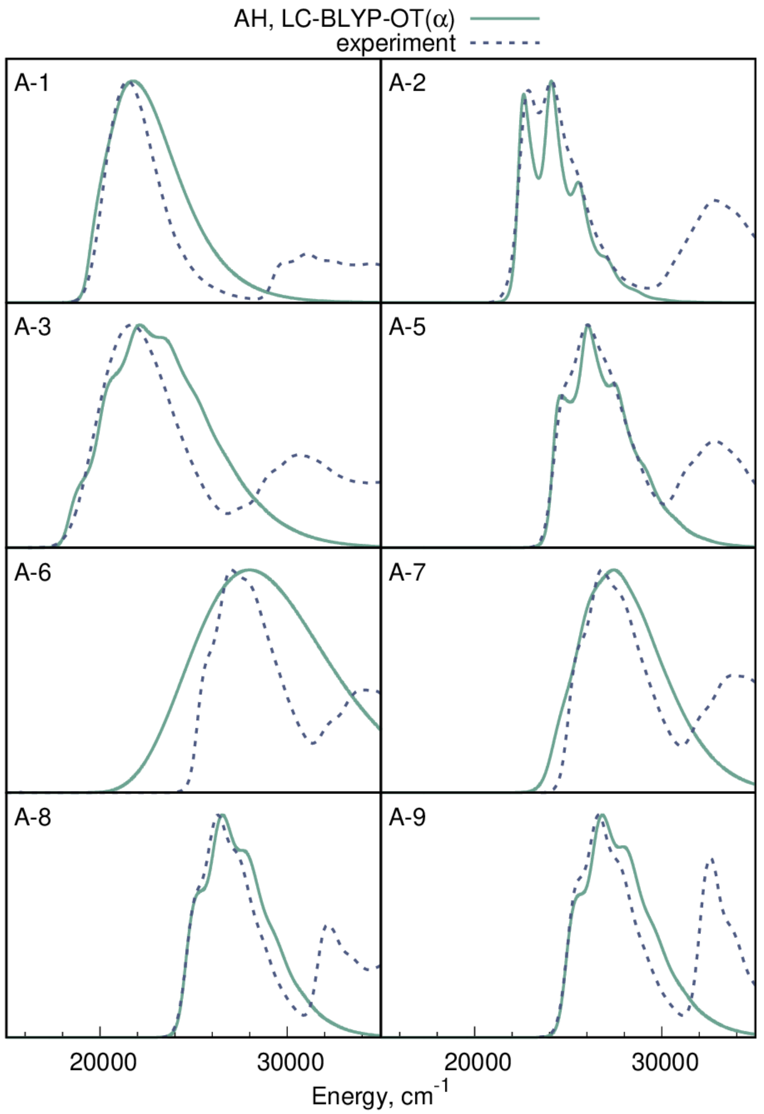

Figure S27: The comparison of the AH model (internal coordinates) with experimental data for series **A**. Shown are the spectra simulated using Gaussian broadening with HWHM = 50 cm^{-1} (and HWHM = 150 cm^{-1} for the structure **A-2**).

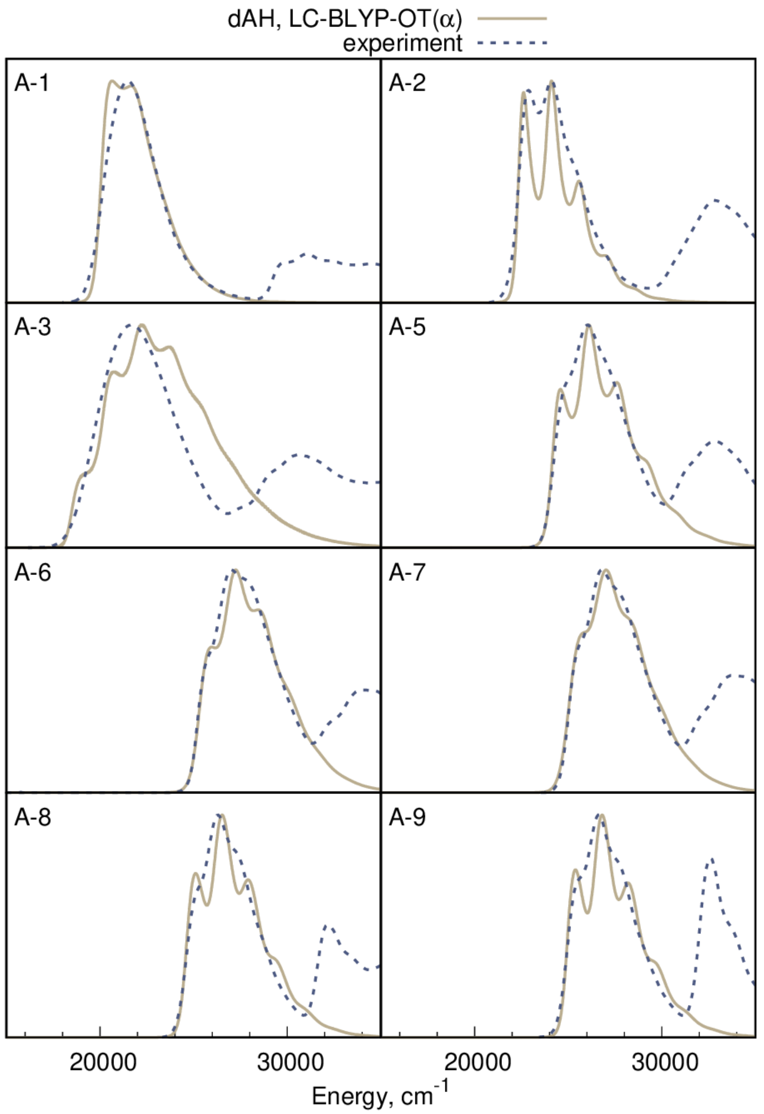

Figure S28: The comparison of the dAH model (internal coordinates) with experimental data for series **A**. Shown are the spectra simulated using empirical Gaussian broadening reproducing FWHM.

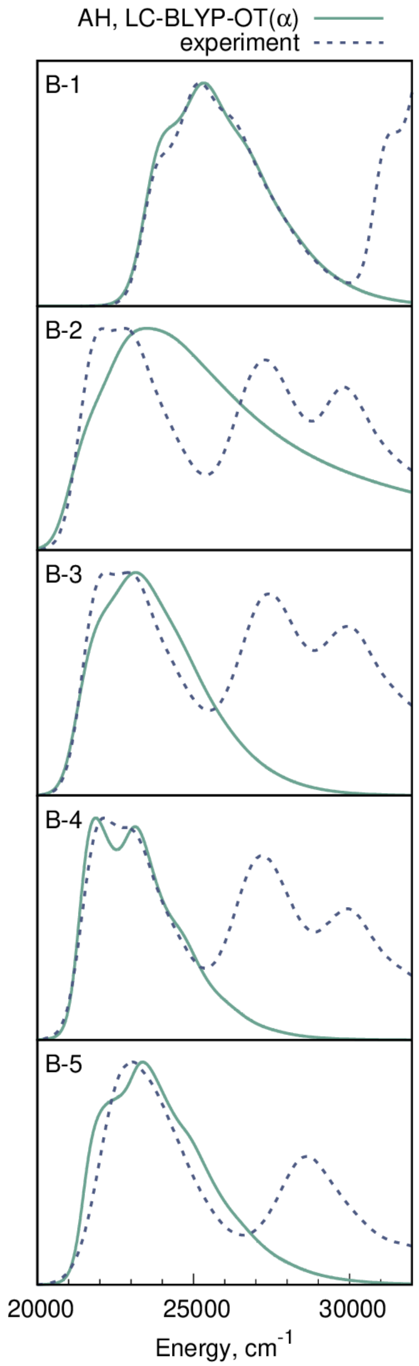

Figure S29: The comparison of the AH model (internal coordinates) with experimental data for series **B**. Shown are the spectra simulated using Gaussian broadening with HWHM = 50 cm^{-1}.

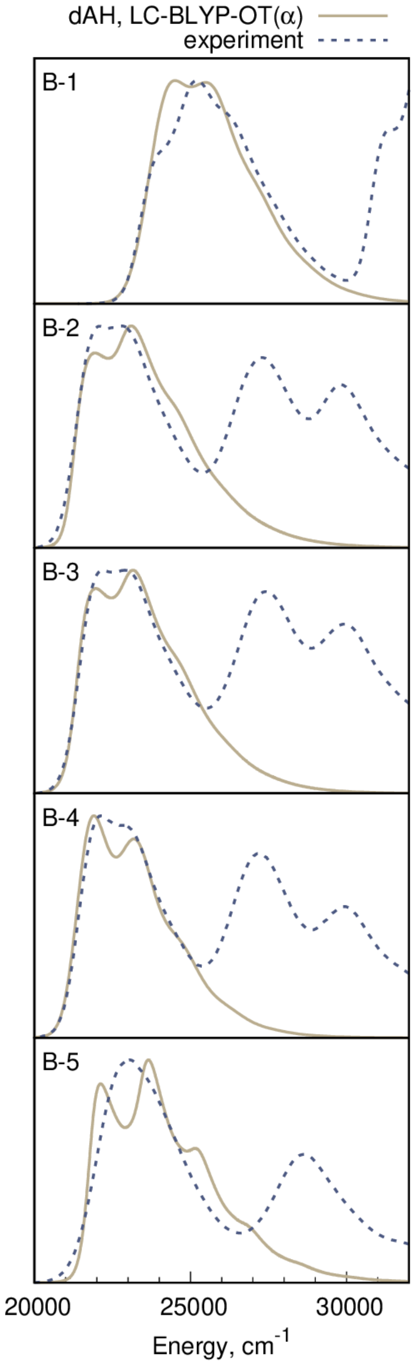

Figure S30: The comparison of the dAH model (internal coordinates) with experimental data for series **B**. Shown are the spectra simulated using empirical Gaussian broadening reproducing FWHM.

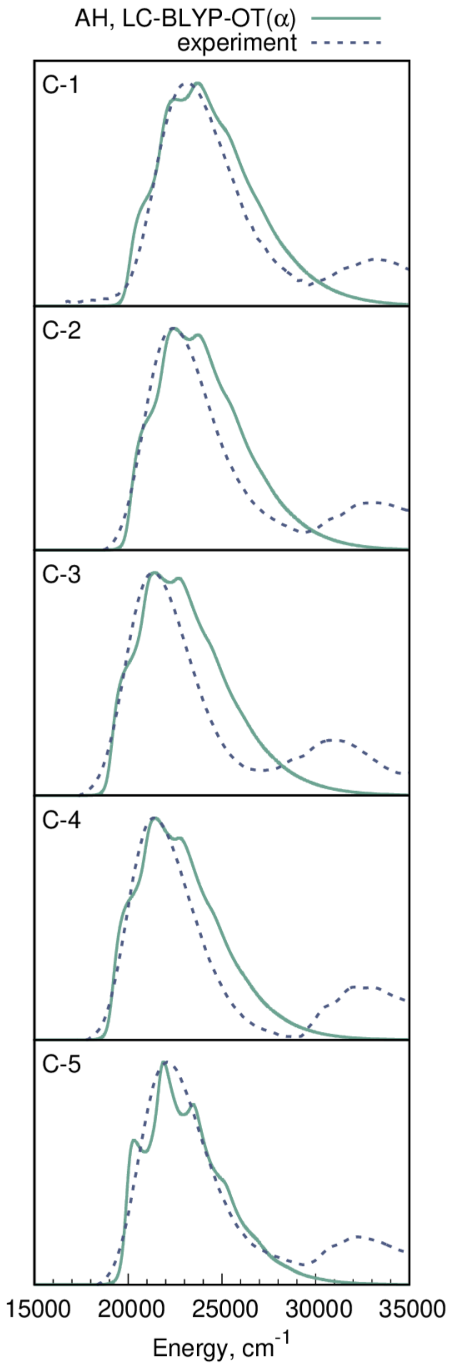

Figure S31: The comparison of the AH model (internal coordinates) with experimental data for series **C**. Shown are the spectra simulated using Gaussian broadening with HWHM = 50 cm^{-1}.

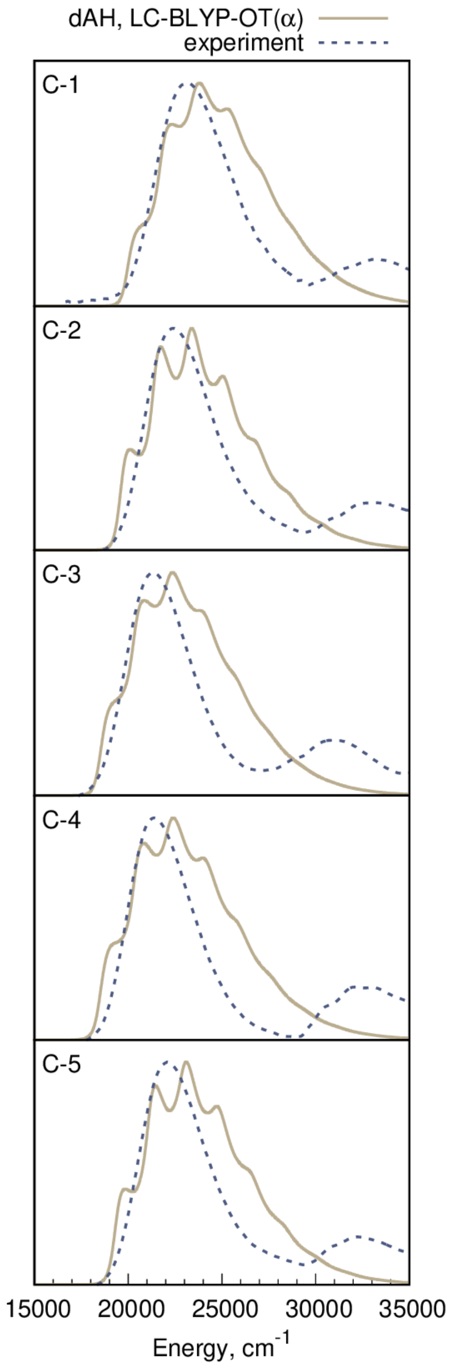

Figure S32: The comparison of the dAH model (internal coordinates) with experimental data for series **C**. Shown are the spectra simulated using Gaussian broadening with HWHM = 50 cm^{-1}.

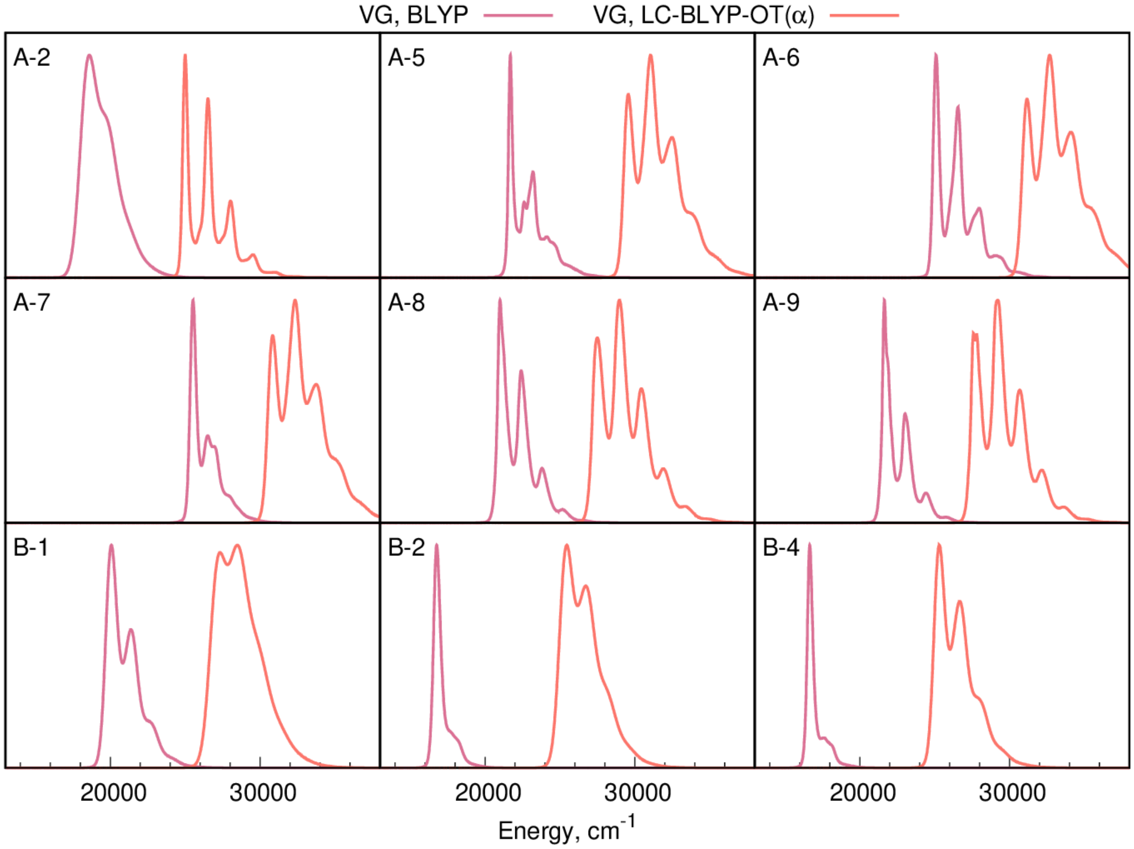

Figure S33: The comparison of BLYP and LC-BLYP-OT(α) for set **A**. Shown are the spectra simulated using the VG aproach with Gaussian broadening (HWHM = 100 cm^{-1}).

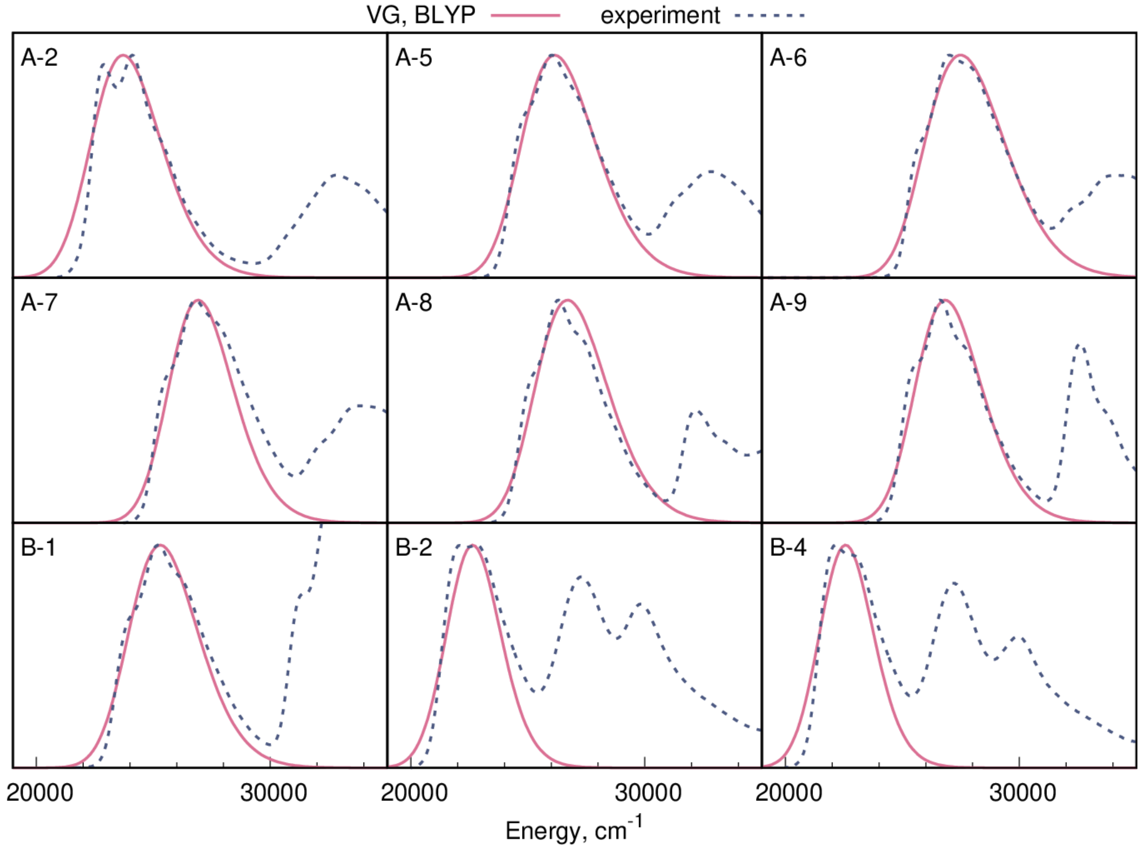

Figure S34: The comparison of the VG model (BLYP functional) with experimental data for chosen molecules. Shown are the spectra simulated using empirical Gaussian broadening reproducing FWHM.

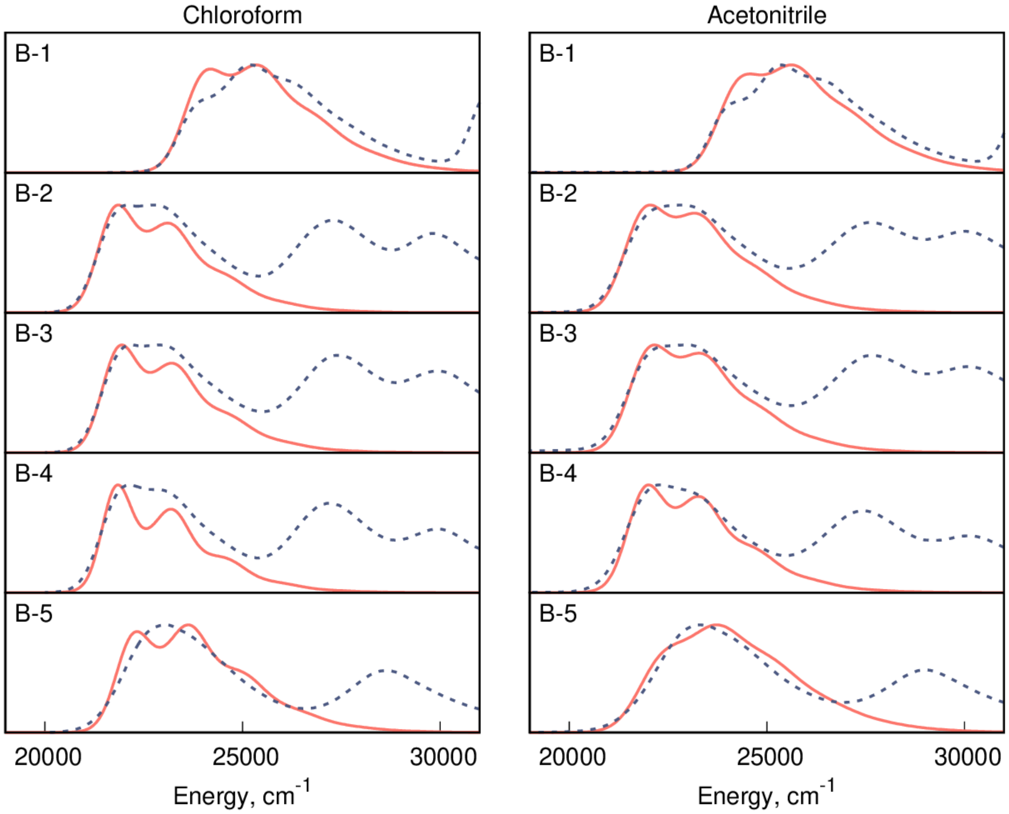

Figure S35: Comparison between the simulated (red solid line) and the experimental (blue dotted line) spectra for five **B** derivatives. The simulations were performed using the VG method and the LC-BLYP-OT(α) DFA with an inhomogeneous broadening estimated using machine learning. See manuscript for details.

References

(S1) Ośmiałowski, B.; Petrusevich, E. F.; Nawrot, K. C.; Paszkiewicz, B. K.; Nyk, M.; Zielak, J.; Jędrzejewska, B.; Luis, J. M.; Jacquemin, D.; Zaleśny, R. Tailoring the nonlinear absorption of fluorescent dyes by substitution at a boron center. *J. Mater. Chem. C* **2021**, *9*, 6225–6233.

(S2) Dziuk, B.; Ośmiałowski, B.; Zarychta, B.; Ejsmont, K.; Chęcińska, L. Symmetric fluoroborate and its boron modification: Crystal and electronic structures. *Crystals* **2019**, *9*.

(S3) Ośmiałowski, B.; Petrusevich, E. F.; Antoniak, M. A.; Grela, I.; Bin Jassar, M. A.; Nyk, M.; Luis, J. M.; Jędrzejewska, B.; Zaleśny, R.; Jacquemin, D. Controlling two-photon action cross section by changing a single heteroatom position in fluorescent dyes. *J. Phys. Chem. Lett* **2020**, *11*, 5920–5925.

(S4) Grabarz, A. M.; Laurent, A. D.; Jędrzejewska, B.; Zakrzewska, A.; Jacquemin, D.; Ośmiałowski, B. The influence of the π-conjugated spacer on photophysical properties of difluoroboranyls derived from amides carrying a donor group. *J. Org. Chem.* **2016**, *81*, 2280–2292.

(S5) Yamaji, M.; Kato, S.-i.; Tomonari, K.; Mamiya, M.; Goto, K.; Okamoto, H.; Nakamura, Y.; Tani, F. Blue fluorescence from BF_2 complexes of N,O-benzamide ligands: Synthesis, structure, and photophysical properties. *Inorg. Chem.* **2017**, *56*, 12514–12519.

(S6) Kaczorowska, M. A.; Ośmiałowski, B. Collision induced dissociation of N-(pyridin-2-yl)-substituted benzo(thio)amides and N-(isoquinolin-1-yl)furan(thiophene)-2-carboxamides and their difluoroboranyl derivatives. *Int. J. Mass Spectrom.* **2018**, *428*, 35–42.

(S7) Duncan, N. C.; Garner, C. M.; Nguyen, T.; Hung, F.; Klausmeyer, K. Electronic effects in the reaction of 1,3-diaryl-1,3-diketones with hydrazinopyridines. *Tetrahedron Lett.* **2008**, *49*, 5766–5769.

(S8) Ono, K.; Yoshikawa, K.; Tsuji, Y.; Yamaguchi, H.; Uozumi, R.; Tomura, M.; Taga, K.; Saito, K. Synthesis and photoluminescence properties of BF_2 complexes with 1,3-diketone ligands. *Tetrahedron* **2007**, *63*, 9354–9358.

(S9) Kolehmainen, E.; Ośmiałowski, B.; Krygowski, T. M.; Kauppinen, R.; Nissinen, M.; Gawinecki, R. Substituent and temperature controlled tautomerism: multinuclear magnetic resonance, X-ray, and theoretical studies on 2-phenacylquinolines. *J. Chem. Soc., Perkin Trans. 2* **2000**, 1259–1266.

(S10) Zakrzewska, A.; Zaleśny, R.; Kolehmainen, E.; Ośmiałowski, B.; Jędrzejewska, B.; Ågren, H.; Pietrzak, M. Substituent effects on the photophysical properties of fluorescent 2-benzoylmethylenequinoline difluoroboranes: A combined experimental and quantum chemical study. *Dyes Pigm.* **2013**, *99*, 957–965.

(S11) Yang, M.-h.; Lin, T.-W.; Chou, C.-C.; Lee, H.-C.; Chang, H.-C.; Lee, G.-H.; Leung, M.-k.; Peng, S.-M. New oligo-α-pyridylamino ligands and their metal complexes. *Chem. Commun.* **1997**, 2279–2280.

(S12) Glotzbach, C.; Kauscher, U.; Voskuhl, J.; Kehr, N. S.; Stuart, M. C. A.; Fröhlich, R.; Galla, H. J.; Ravoo, B. J.; Nagura, K.; Saito, S.; Yamaguchi, S.; Würthwein, E.-U. Fluorescent modular boron systems based on NNN- and ONO-tridentate ligands: Self-assembly and cell imaging. *J. Org. Chem.* **2013**, *78*, 4410–4418.
